# Unimolecular Self-Assembled Hemicyanine–Oleic Acid Conjugate Acts as a Novel Succinate Dehydrogenase Inhibitor to Amplify Photodynamic Therapy and Eliminate Cancer Stem Cells

**DOI:** 10.34133/research.0223

**Published:** 2023-09-06

**Authors:** Qiang Wang, Tian Yang, Shiyou Li, Chen Xu, Chong Wang, Yuxuan Xiong, Xing Wang, Jiangling Wan, Xiangliang Yang, Zifu Li

**Affiliations:** ^1^ National Engineering Research Center for Nanomedicine, College of Life Science and Technology, Huazhong University of Science and Technology, Wuhan 430074, P. R. China.; ^2^ Key Laboratory of Molecular Biophysics of Ministry of Education, College of Life Science and Technology, Huazhong University of Science and Technology, Wuhan 430074, P. R. China.; ^3^ Hubei Key Laboratory of Bioinorganic Chemistry and Materia Medical, Huazhong University of Science and Technology, Wuhan 430074, P. R. China.; ^4^ Hubei Engineering Research Center for Biomaterials and Medical Protective Materials, Huazhong University of Science and Technology, Wuhan 430074, P. R. China.; ^5^ Hubei Bioinformatics and Molecular Imaging Key Laboratory, College of Life Science and Technology, Huazhong University of Science and Technology, Wuhan 430074, P. R. China.

## Abstract

Photodynamic therapy with reactive oxygen species production is a prospective treatment to combat cancer stem cells (CSCs). However, the innate drawbacks, including short lifetime and diffusion distance of reactive oxygen species and hypoxia within solid tumors, have become bottlenecks for clinical applications of photodynamic therapy. Here, we develop a mitochondria-targeting hemicyanine–oleic acid conjugate (CyOA), which can self-assemble into supramolecular nanoparticles (NPs) without any exogenous excipients. CyOA is also shown for targeting the mitochondrial complex II protein succinate dehydrogenase to inhibit oxidative phosphorylation and reverse tumor hypoxia, resulting in 50.4-fold higher phototoxicity against breast cancer stem cells (BCSCs) compared to SO_3_-CyOA NPs that cannot target to mitochondria. In 4T1 and BCSC tumor models, CyOA NPs achieve higher tumor inhibition and less lung metastasis nodules compared to the clinically used photosensitizer Hiporfin. This study develops a self-assembled small molecule that can serve as both oxidative phosphorylation inhibitor and photosensitizer for eradication of CSCs and treatment of solid tumors.

## Introduction

Cancer stem cells (CSCs), also regarded as tumor-initiating cells or tumor-repopulating cells (TRCs), have been considered the Achilles heel of cancer therapy, as these small fraction tumor cells have been accused as the main culprit in tumor initiation, progression, metastasis, chemoresistance, and recurrence [1]. With the growing evidence that reactive oxygen species (ROS) can govern CSC self-renewal and stemness [2], many conventional redox regulators, such as all-*trans* retinoic acid [3], salinomycin [4], and doxycycline [5], have been leveraged to eliminate CSCs. However, compared to chemotherapeutic agents that produce limited endogenous ROS by intrinsic biological pathways, photodynamic therapy (PDT) shows great potential in abolishing CSCs by inducing ROS burst through photochemical reactions [6–8].

Nevertheless, the inherent high oxygen reliance nature of PDT weakens its antitumor potency, especially for CSCs that are deeply anchored in the hypoxic regions of solid tumors [9,10]. To overcome this challenge, 2 approaches have been introduced to overcome tumor hypoxia. One is the oxygen-supply strategy, such as directly delivering oxygen to tumor tissues (e.g., perfluorocarbon [11], hemoglobin [12], and red blood cells[13]) or catalyzing endogenous H_2_O_2_ to O_2_ (e.g., MnO_2_ [14], Prussian blue [15], and catalase [16]). This method faces several challenges, including ineffective loading and rapid leakage of O_2_ for O_2_ carriers, and limited intratumoral H_2_O_2_ level for O_2_ generators. The alternative method is to develop O_2_ economizer to inhibit oxidative phosphorylation (OXPHOS) through active drugs, such as atovaquone [17], nitric oxide [18,19], and iridium(III) complexes [20]. For instance, Fan et al. [17] developed a nano-drug delivery system to co-deliver photosensitizer and atovaquone, while Li et al. [21] tailored a photosensitizer–OXPHOS inhibitor conjugate. However, complex preparation techniques of multiple drug co-delivery systems and poor pharmacokinetic profiles of photosensitizer–oxygen regulator conjugates due to their high molecular weight limit these O_2_ economizers for clinical translation. Therefore, it is urgent to develop simple and efficient PDT to overcome tumor hypoxia.

**Fig. 1.**
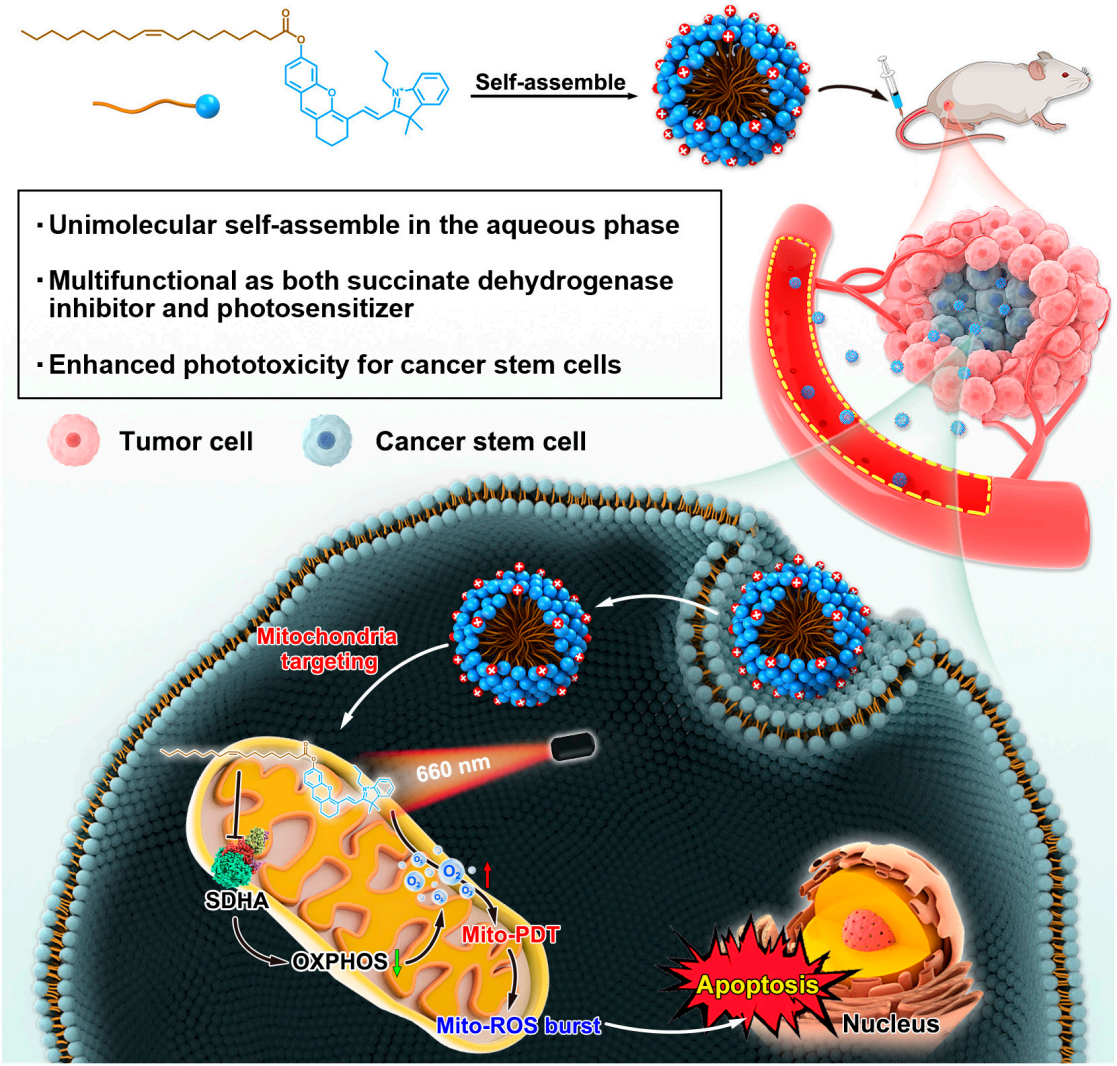
Schematic illustration of self-assembled CyOA NPs to eliminate CSCs by simultaneously suppressing oxygen consumption and inducing mitochondrial PDT.

Over the past decade, advanced nanomedicine for cancer treatment has attracted tremendous interests [22–26]. Among them, pure drug self-assembly systems have been extensively pursued for their simple preparation process and ultrahigh drug-loading efficacy [27–29]. Hemicyanine scaffold dyes, with near-infrared emission (>650 nm), have been widely applied as smart fluorescent probes and photosensitizers [30,31]. Unsaturated aliphatic chains are frequently used to facilitate the formation of nanoassemblies for hydrophobic drugs, such as docetaxel [27], losartan [28], and chlorine e6 [29]. In this study, we introduced oleic acid to conjugate hemicyanine scaffold dyes (CyOH or SO_3_-CyOH), which can self-assemble into supramolecular nanoparticles (NPs) without any exogenous excipients. One of these supramolecular NPs composed of CyOH–oleic acid conjugates is positively charged and named CyOA NPs, while the other formed from SO_3_-CyOH–oleic acid conjugates is negative and designated SO_3_-CyOA NPs. As shown in Fig. 1, self-assembled CyOA NPs implemented mitochondrial PDT (Mito-PDT) to address the short lifetime and diffusion distance of ROS, enabling maximum cancer cell killing capacity. Besides, for the first time, we unexpectedly found that CyOA, rather than SO_3_-CyOA, could target the mitochondrial complex II protein succinate dehydrogenase (SDHA) to inhibit OXPHOS and reverse tumor hypoxia. As a consequence, CyOA NPs achieved strikingly enhanced phototoxicity against breast cancer stem cells (BCSCs) both in vitro and in vivo. This work provides an exquisite example for developing multifunctional self-assembled agents to eliminate CSCs by congregating O_2_-conserving and ROS-bursting functions in a single molecule.

## Results

### Synthesis and characterization of SO_3_-CyOA and CyOA

**Fig. 2.**
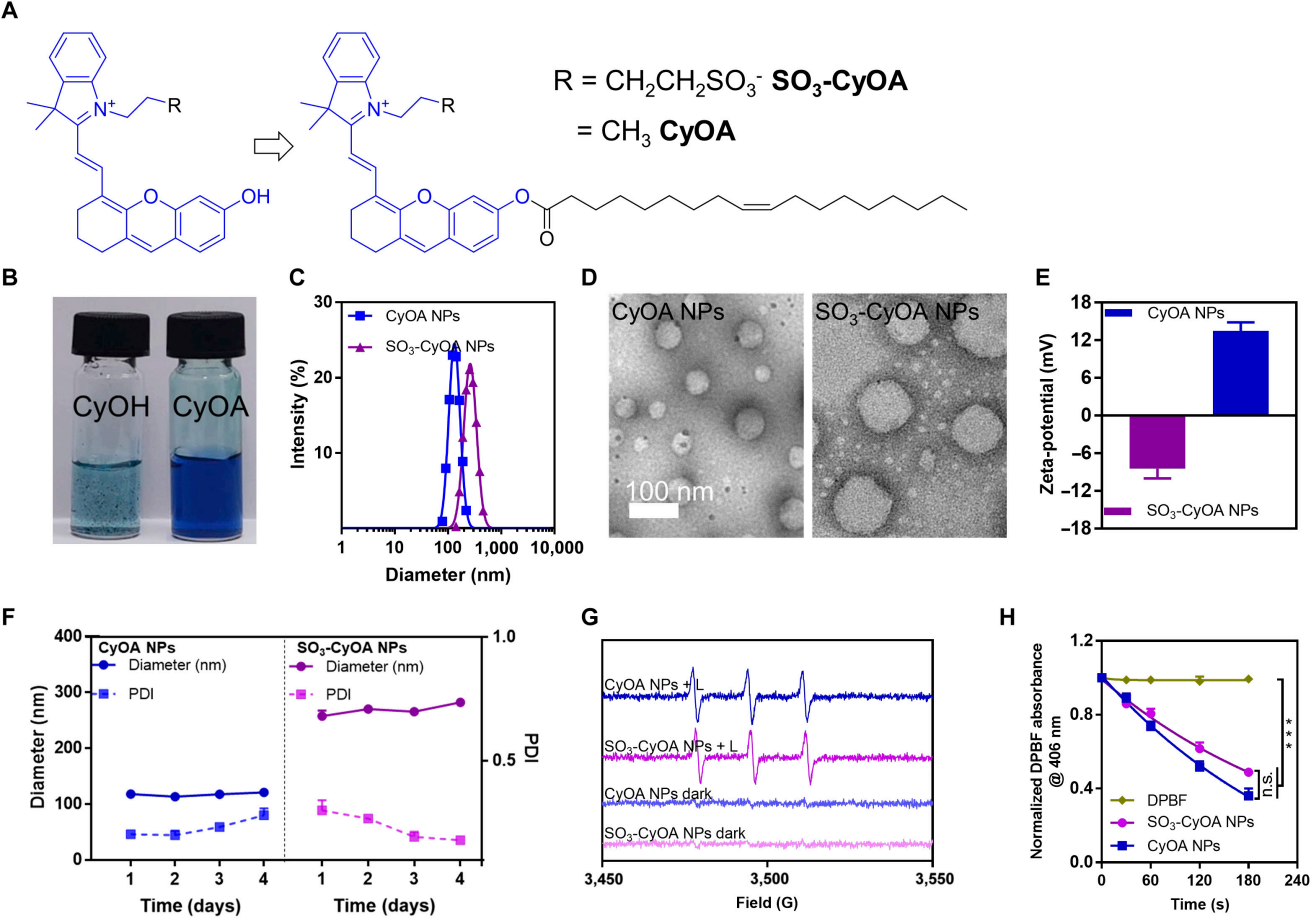
Unsaturated aliphatic chain modification facilitated the self-assembly of oleic acid–hemicyanine conjugates. (A) Chemical structures of CyOA and SO_3_-CyOA. (B) Images of CyOH and CyOA dispersed in water. (C) Particle size distribution of CyOA NPs and SO_3_-CyOA NPs. (D) TEM images of CyOA NPs and SO_3_-CyOA NPs. The scale bar is 100 nm. (E) Zeta potential of CyOA NPs and SO_3_-CyOA NPs (*n* = 3). (F) Storage stability of CyOA NPs and SO_3_-CyOA NPs in deionized water (*n* = 3). (G) Electron paramagnetic resonance (EPR) signals of ^1^O_2_ generated by CyOA NPs and SO_3_-CyOA NPs with or without 660-nm laser irradiation. (H) Quantification of DPBF absorbance at 406 nm in the presence of CyOA NPs and SO_3_-CyOA NPs under 660-nm laser irradiation (*n* = 3). Data represent mean ± SEM, ***P < 0.001; n.s. stands for not significant.

As unsaturated aliphatic chain can promote self-assembly of the water-insoluble molecules [27], biocompatible oleic acid was used to conjugate the hydroxyl group of hemicyanine (Fig. 2A). Scheme S1 presents the synthetic route of CyOA and SO_3_-CyOA. Commercially available IR780 or IR783 was first reacted with resorcinol to afford CyOH or SO_3_-CyOH, followed by esterification with oleic acid to give CyOA and SO_3_-CyOA, respectively. All final compounds were characterized by high-resolution mass spectrometry (HRMS) (Fig. S1) and nuclear magnetic resonance spectra (^1^H NMR and ^13^C NMR) (Figs. S2 and S3). Spectroscopic characteristics indicated that the maximum absorption wavelengths of both CyOA and SO_3_-CyOA were blue-shifted (λ_ab_ = 600 nm) compared with that of CyOH and SO_3_-CyOH (λ_ab_ = 670 nm) (Fig. S4). Since the electron-donating capacity of the hydroxyl group was partially neutralized, the fluorescence emission of CyOA and SO_3_-CyOA was negligible compared with CyOH and SO_3_-CyOH (λ_em_ = 740 nm) due to the intramolecular charge transfer effect (Fig. S5). However, a nearly 5-fold fluorescence “turn-on” enhancement at 720 nm was observed for both CyOA and SO_3_-CyOA after co-incubation with porcine liver esterase (100 U/ml) for 12 h. These changes indicated the esterase-induced cleavage of ester linkage and subsequently recovery of near-infrared region (NIR) fluorescence signals, providing a primer for intracellular and in vivo drug tracking.

### Preparation and characterization of SO_3_-CyOA NPs and CyOA NPs

We next examined whether CyOA and SO_3_-CyOA could self-assemble by using reprecipitation protocol. Upon exposure to a poor solvent (i.e., water), the hemicyanine–oleic acid conjugates underwent self-assembly to form transparent solutions rather than precipitates (Fig. 2B). Transmission electron microscopy (TEM) revealed that both CyOA and SO_3_-CyOA formed spherical NPs in water (Fig. 2D). Dynamic light scattering (DLS) measurements further confirmed that CyOA NPs exhibited a positive surface charge, whereas SO_3_-CyOA NPs showed a negative surface charge (Fig. 2E). The average hydrodynamic diameters of CyOA NPs and SO_3_-CyOA NPs were around 114 nm and 235 nm, respectively (Fig. 2C). Such size variation may be ascribed to changes in electrostatic interaction. Stability test demonstrated that CyOA NPs remained stable for 4 days upon incubation in different media, including deionized water (Fig. 2F), saline, and saline containing 20% fetal bovine serum (FBS) (Fig. S6). Remarkably, without any exogenous excipients, the solution of CyOA NPs remained transparent even up to 30 mM. Overall, a pure drug self-assembly system by conjugating payload with biocompatible oleic acid holds great potential for clinical translation, as the toxicity problems associated with excipients are averted.

### Singlet oxygen analysis in solution

We employed 2,2,6,6-tetramethylpiperidine (TEMP) and 1,3-diphenylisobenzofuran (DPBF) reagents to investigate the ^1^O_2_ generating ability of photosensitizer in solution. As shown in Fig. 2G, upon irradiation of CyOA NPs and SO_3_-CyOA NPs, characteristic peaks (Temp−^1^O_2_ adducts) appeared in the electron paramagnetic resonance (EPR) spectra, whereas no peaks were observed in the absence of irradiation, indicating laser irradiation-induced ^1^O_2_. The attenuated absorption of DPBF further demonstrated ^1^O_2_ production after irradiation of the oleic acid–hemicyanine conjugates (Fig. S7). Figure 2H reveals that there is no difference in the capacity of CyOA NPs and SO_3_-CyOA NPs to generate ^1^O_2_ under 660 nm laser irradiation in solution.

### Cellular uptake and mitochondrial targeting

**Fig. 3.**
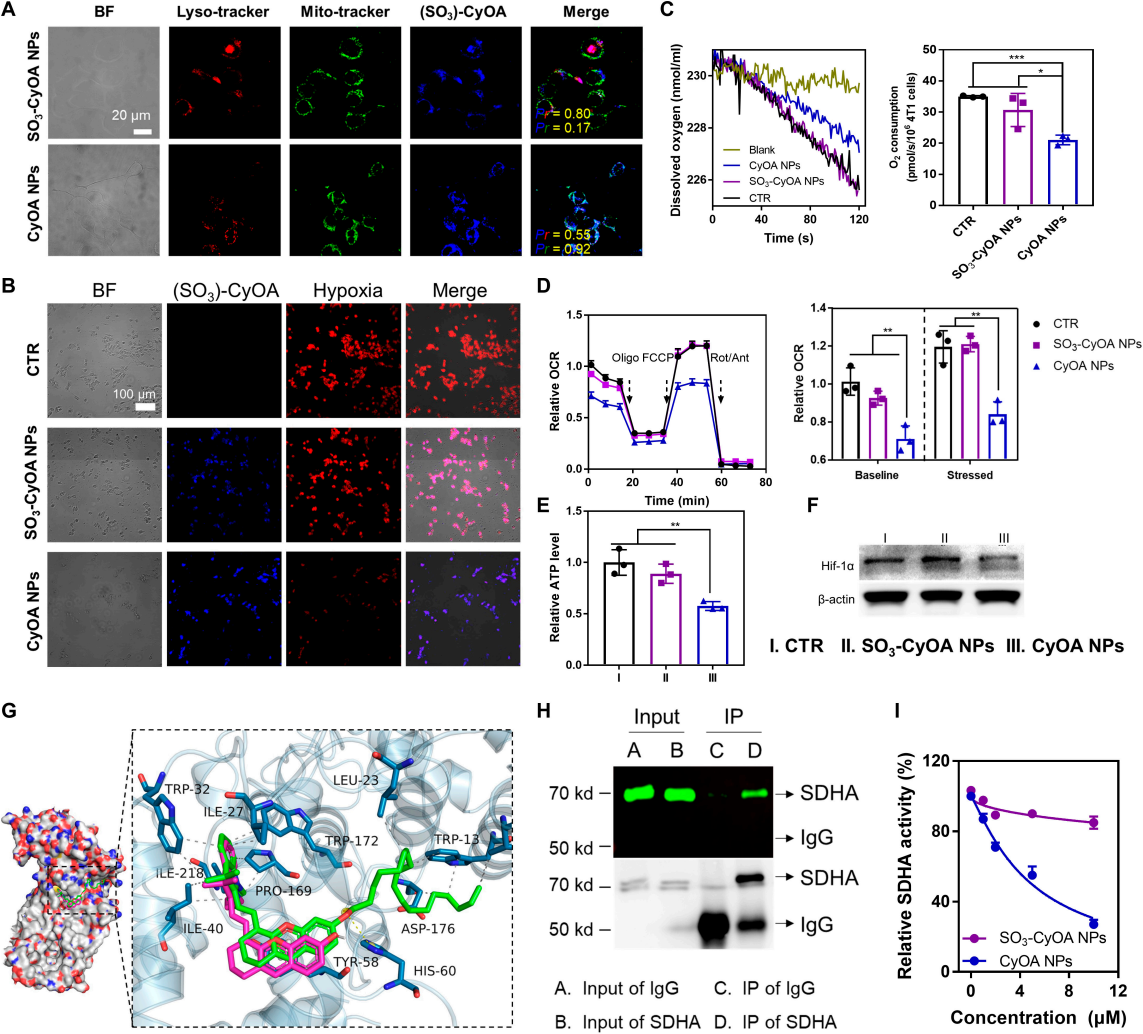
CyOA binds SDHA to suppress OXPHOS and alleviate hypoxia. (A) CLSM images of intracellular distribution of CyOA NPs and SO_3_-CyOA NPs. The scale bar is 20 μm. (B) Determination of cellular hypoxia in 4T1 cells after treatment with SO_3_-CyOA NPs (20 μM) or CyOA NPs (2 μM). The scale bar is 100 μm. (C) Representative traces of dissolved O_2_ content in cell culture medium after 4T1 cells with different treatments using Clark oxygen electrode. Histograms representing the oxygen consumption rate (OCR) of 4T1 cells in different groups (*n* = 3). (D) OCR in 4T1 cells in the absence or presence of SO_3_-CyOA NPs (20 μM) or CyOA NPs (2 μM) and determined using the Seahorse extracellular flux analyzer. Histograms representing OCR before (baseline) and after (stressed) olig and FCCP addition (*n* = 3). Olig: oligomycin; Rot: rotenone; Ant: antimycin A. (E) Relative intracellular ATP levels after different treatments (*n* = 3). (F) Western blot analysis of Hif-1α protein in 4T1 cells. (G) Binding mode of CyOH (magenta stick) and CyOA (green stick) with SDHA (PDB ID: 2FBW). (H) SDHA pull-down assay in CyOA-treated 4T1 cell lysates. IgG: immunoglobulin G; IP: immunoprecipitation. (I) Dose–effect curve of CyOA NPs and SO_3_-CyOA NPs on intracellular SDHA activity (*n* = 3). Data represent mean ± SEM, *P < 0.05; **P < 0.01; ***P < 0.001.

Positively charged substances enhance cellular uptake as well as accumulation in mitochondria [32]. Flow cytometry analysis showed that the cellular uptake of 2 μM positive-charged CyOA NPs and 20 μM negative-charged SO_3_-CyOA NPs was comparable; therefore, the corresponding concentrations of CyOA NPs and SO_3_-CyOA NPs were used for the next cellular experiments (Fig. S8). A confocal laser scanning microscope (CLSM) was used to explore the intracellular location of CyOA NPs in 4T1 cells. As shown in Fig. 3A, we observed that there was clear co-localization of blue-colored CyOA with green-colored Mito-Tracker. Pearson’s correlation coefficient between mitochondrion and CyOA was 0.92, far larger than that of lysosome and CyOA (0.55). However, due to the negative surface charge, more SO_3_-CyOA NPs were located in lysosomes than in mitochondria, which is in contrast to CyOA NPs. The same conclusion was obtained when dyes were quantified on isolated mitochondria, where CyOA was about 10-fold higher than that of SO_3_-CyOA (Fig. S9). Thus, tailoring CyOA NPs to target mitochondria addressed the short lifetime and diffusion distance of ROS.

### Inhibition of OXPHOS and alleviation of hypoxia

Noteworthily, for the first time, we unexpectedly found that indole-based cationic dye, CyOA, could efficiently inhibit OXPHOS and reverse tumor hypoxia. Intracellular hypoxia level was assayed by hypoxia/oxidative stress detection kit ROS-ID, whose fluorescence could “turn-on” because the nitro group of ROS-ID was reduced to the amino group under hypoxic conditions. As shown in Fig. 3B, CLSM images showed that compared with control and the SO_3_-CyOA NP-treated groups, cells in the CyOA NP-treated group showed much dimmer red fluorescence, demonstrating a much lower hypoxia degree. Flow cytometry analysis further verified that red fluorescence in the CyOA NP-treated group was nearly 3-fold lower relative to the control and SO_3_-CyOA NP-treated groups (Fig. S10). We further evaluated the impact of CyOA NPs on mitochondrial oxygen consumption rate (OCR) by using a Clark oxygen electrode and a Seahorse XF analyzer. As illustrated in Fig. 3C, the rate of decline in dissolved O_2_ concentration of the SO_3_-CyOA NP-treated group was almost identical to untreated cells, but dramatically higher than that of the CyOA NP-treated group. Quantitative analysis revealed that dissolved O_2_ in the cell culture medium was reduced by nearly 40% after 6 h of treatment with 2 μM CyOA NPs. The effect of CyOA NPs on cellular OXPHOS was then precisely assessed using the Seahorse XF analyzer. As shown in Fig. 3D, compared to SO_3_-CyOA NPs with no effect on OCR, significant decreases in both basal and maximal respiration were observed with 2 μM CyOA NPs treatment, suggesting that CyOA can efficiently inhibit OXPHOS. Intracellular adenosine triphosphate (ATP) assay also verified that CyOA NPs (2 μM) significantly blocked ATP synthesis (Fig. 3E). Moreover, we found that CyOA NPs could remarkably downregulate the expression of Hif-1α in hypoxic cells compared to SO_3_-CyOA NP-treated and control groups (Fig. 3F). Altogether, such an incidental finding, that the photosensitizer itself can act as an OXPHOS inhibitor and downregulate Hif-1α expression, provides a potential novel paradigm to develop O_2_ economizer for overcoming hypoxia in PDT.

### Validation of specific SDHA protein binding

Previous research has documented that IR-26, a heptamethine cyanine dye, can directly target SDHA to inhibit mitochondrial complex II activity [33], and novel SDHA inhibitors typically introduce an indole group [34]. Therefore, we speculated whether CyOA inhibited OXPHOS by targeting SDHA. As all reported SDHAs are highly reserved [35], we selected avian (*Gallus gallus*) respiratory complex II with carboxin binding pocket [Protein Data Bank (PDB) code: 2FBW] for molecular docking studies. As shown in Fig. 3G, both CyOA and CyOH were embedded in the active pocket of SDHA protein and formed a π-stacking interaction with the residue of TYR-58D. The carbonyl oxygen in CyOA creates a salt bridge with HIS-60D, and its indole group extended toward the entrance of the SDHA binding site and forms hydrophobic interactions with PRO-169B, TRP-172B, ILE-218B, ILE-27C, TRP-32C, and ILE-40C. The lowest binding energy values of CyOA and CyOH are −7.9 kcal/mol and −8.4 kcal/mol, respectively, which are less than the standard reference compound Carboxin (−5.5 kcal/mol). Thus, molecular docking studies suggested that both CyOH and CyOA possessed a potent interaction with SDHA. Next, pull-down assays were performed to validate the interaction between CyOA and SDHA. As shown in Fig. 3H, SDHA antibody can pull-down proteins labeled with NIR fluorescence in CyOA-treated cell lysates, indicating that CyOA directly binds with the mitochondrial complex II protein SDHA. Intracellular SDHA activity assay also indicated that CyOA dose-dependently decreased SDHA activity, with nearly 80% reduction upon treatment with 10 μM of CyOA for 8 h (Fig. 3I). Therefore, these data suggest that CyOA can directly target the mitochondrial complex II protein SDHA and subsequently disturb mitochondrial OXPHOS function to alleviate hypoxia.

### Intracellular total ROS and Mito-ROS production

**Fig. 4.**
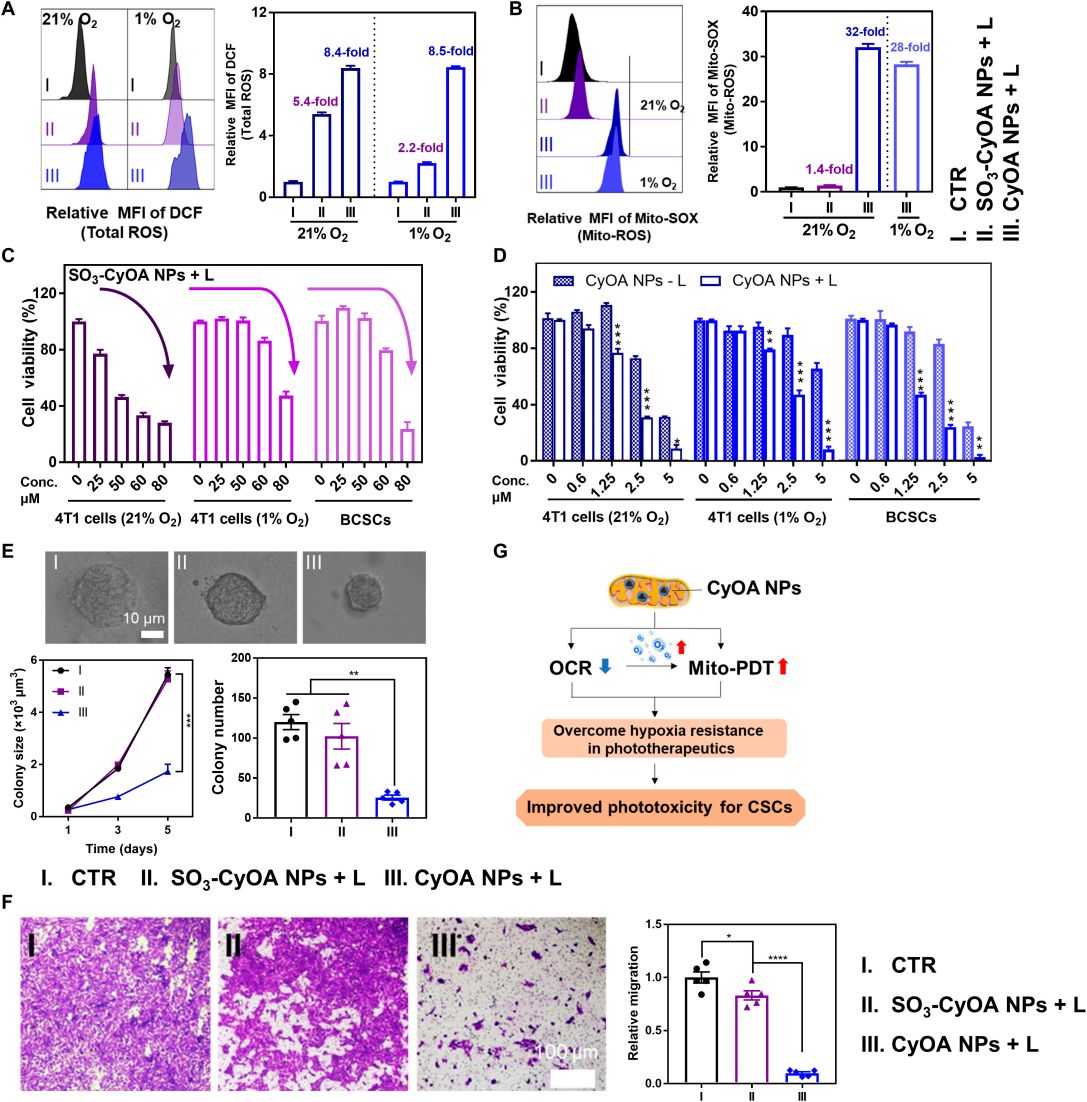
Attenuated hypoxia resulted in enhanced phototoxicity of CyOA NPs against BCSCs. (A) Flow cytometry analysis of total ROS after various treatments using the fluorescent probe DCFDA (*n* = 3). (B) Flow cytometry analysis of Mito-ROS by Mito-SOX Red staining (*n* = 3). (C) Phototoxicity of SO_3_-CyOA NPs against normoxic and hypoxic 4T1 cells, as well as BCSCs (*n* = 3). (D) Phototoxicity and dark toxicity of CyOA NPs against normoxic and hypoxic 4T1 cells, as well as BCSCs (*n* = 3). (E) Representative images of 4T1 TRCs, and the scale bar is 10 μm; colony size–day curves (*n* = 15) and quantified colony numbers on day 5 (*n* = 5) after different treatments. (F) Migration of 4T1 cells with different treatments in transwell migration assays. Histograms representing relative migration fields (*n* = 5). (G) Schematic illustration of CyOA NPs inhibiting cellular respiration to overcome hypoxia in PDT. Data represent mean ± SEM, *P < 0.05; **P < 0.01; ***P < 0.001; ****P< 0.0001.

Then, the photodynamic properties were evaluated in cellular levels. 2',7'-Dichlorodihydrofluorescein diacetate (DCFDA) and MitoSOX Red were used to detect total ROS and mitochondrial ROS (Mito-ROS), respectively. As shown in Fig. 4A, as compared to limited ROS under hypoxic conditions, SO_3_-CyOA NP-treated cells exhibited higher levels of total ROS under a normoxic environment. In stark contrast, CyOA NP-treated cells exhibited similarly potency to generate total ROS under both normoxic and hypoxic conditions. These results confirmed that CyOA NPs conserved endogenous O_2_ for subsequent PDT. Likewise, the fluorescence intensity of Mito-SOX red in the CyOA NP-treated group reached 32-fold that of PBS group, while SO_3_-CyOA NP-treated cells yielded limited Mito-ROS (Fig. 4B). Furthermore, despite under hypoxic conditions, the CyOA NP-treated group showed efficient Mito-ROS generation, with a 28-fold higher red fluorescence intensity than the PBS group. Collectively, these results demonstrated that CyOA NPs can overcome hypoxia resistance and subsequently initiate Mito-ROS burst upon photoirradiation.

### In vitro phototoxicity against 4T1 cells

Encouraged by the mitochondrial targeting as well as cellular respiration inhibition properties of CyOA NPs, we hypothesized that CyOA NPs could potently achieve phototoxicity under both normoxic and hypoxic conditions. The significant difference of phototoxicity between CyOA NPs and SO_3_-CyOA NPs might be partially ascribed to more CyOA NPs located in mitochondria (Fig. 4C and D). As shown in Table S1, compared with the IC_50_ value of SO_3_-CyOA NPs under hypoxia (IC_50_: 79.3 μM), which was remarkably larger than that under normoxia (IC_50_: 45.7 μM), CyOA NPs displayed similar phototoxicity under normoxia (IC_50_: 1.9 μM) and hypoxia (IC_50_: 2.1 μM). As blocking OXPHOS affected the proliferation of tumor cells, CyOA NPs showed some degree of dark toxicity, with IC_50_ values of 4.1 μM and 6.4 μM on normoxic and hypoxic 4T1 cells, respectively (Fig. 4D). Therefore, these results collectively validated that using self-assembled CyOA NPs to enable O_2_-conserving mitochondria-targeted PDT can indeed solve inherent drawbacks of traditional PDT, showing significant potential for clinical applications.

### In vitro phototoxicity against BCSCs

Hypoxia harbors CSCs [1], while mitochondria and Mito-ROS drive CSC functional traits [36]. Thus, we hypothesized that O_2_-economical mitochondrial-targeted CyOA NPs have high potential to inhibit CSCs. Two methods were introduced to evaluate the antiproliferative potency of CyOA NPs on BCSCs in vitro. One is the gold standard assay of CSCs, known as the mammospheres formation test. [5] We first plated 4T1 cells in ultra-low attachment culture dishes with CSC culturing medium. After 5 days, collected mammospheres were used for cytotoxicity assay with the CCK-8 kit. As shown in Fig. 4C and D, phototoxicity studies revealed that the drug resistance of mammospheres was sensitive to CyOA NPs rather than to SO_3_-CyOA NPs, with IC_50_ values of 1.4 μM and 70.6 μM, respectively. The latter approach aims to evaluate TRCs, a population of cells that were selected and enriched by 3-dimensional (3D) soft fibronectin gels and exhibited high tumorigenicity [37]. As shown in Fig. 4E, compared to SO_3_-CyOA NPs having almost no inhibitory effect on 4T1 TRCs, upon irradiation, 2 μM CyOA NPs remarkably inhibited the growth of 4T1 TRCs, with colony number decreased from 120 to 26 (by 78%) and the colony size diminished from 5.4 × 10^3^ μm^3^ to 1.7 × 10^3^ μm^3^ (by 69%). The proportion of CSCs in vitro was further analyzed by flow cytometry with CSC markers, such as CD133^+^ (Fig. S11) and CD44^+^CD24^−^ (Fig. S12). The CyOA NPs + L group always showed lower CSC rates compared to the control group, with CD133^+^ and CD44^+^ CD24^−^ population cells decreased from 7.2% to 4.3% (by 40%) and 10.9% to 4.1% (by 62%), respectively. Furthermore, considering that CSCs are essential for tumor invasion and metastasis, the impact of CyOA NPs on migration was investigated using transwell migration assay (Fig. 4F). Upon irradiation, over 90% of cellular migration was suppressed in the 2 μM CyOA NP-treated group, far larger than the 20% inhibition rate in the 20 μM SO_3_-CyOA NP-treated group. Together, these results demonstrated that CyOA NPs achieved strikingly enhanced phototoxicity against BCSCs by overcoming hypoxia and subsequently Mito-ROS burst under light (Fig. 4G).

### Pharmacokinetic and biodistribution of CyOA NPs

Next, the pharmacokinetic profile of supramolecular NPs was evaluated. The plasma concentration–time profiles of CyOH and CyOA NPs are presented in Fig. S13a and the pharmacokinetic parameters are summarized in Table S2. Compared to the rapid clearance of CyOH from blood, self-assembled CyOA NPs had an obviously longer circulation time, with half-life (*t*_1/2_) and area under curve (AUC) increased by 1.7-fold and 15.8-fold, and clearance rate (CL) decreased by 10-fold. Afterwards, 4T1 tumor-bearing BALB/C mice were used to evaluate the biodistribution of the self-assembled NPs. As shown in Fig. S13B to E, CyOA NPs rapidly accumulated in tumor parenchyma and remained at a high level within 24 h. The *ex vivo* images also revealed a higher retention of hemicyanine in tumors in the CyOA NP group than in the free CyOH group. After 24 h of administration, the fluorescence intensity of tumors in the CyOA NPs group was nearly 2-fold higher than that of the free CyOH group. The above results highlighted the key role of the long unsaturated aliphatic chain in contributing to the favorable pharmacokinetics of hemicyanine and enhancing its permeability and retention in tumor tissues.

### In vivo antitumor efficacy in the subcutaneous 4T1 tumor model

**Fig. 5.**
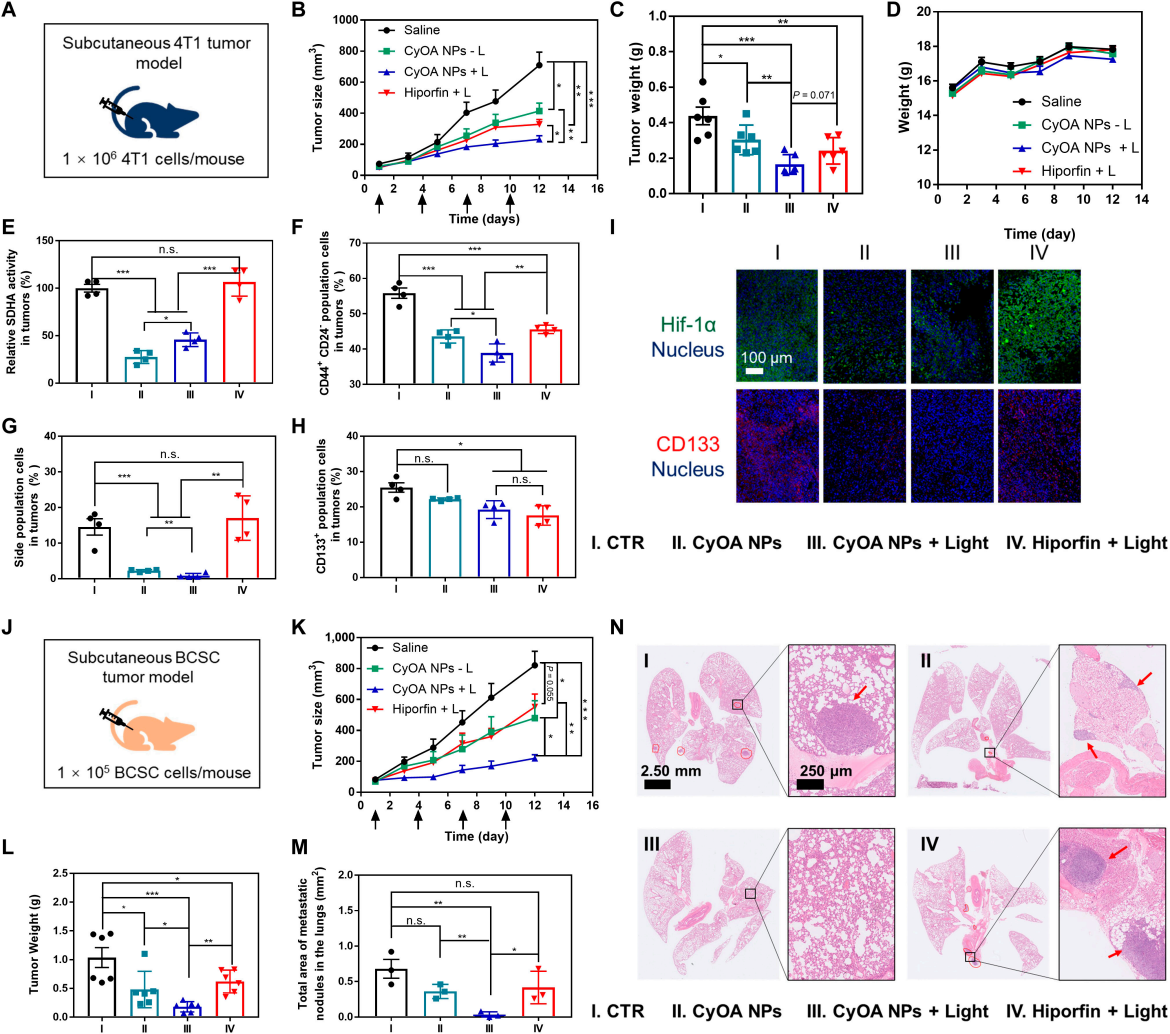
CyOA NPs suppressed tumor growth in subcutaneous 4T1 and BCSC tumor models. (A to I) Subcutaneous 4T1 tumor model. (J to N) Subcutaneous BCSC tumor model. (A) Schematic illustration for the method to construct the subcutaneous 4T1 tumor model. (B) Tumor growth curves (*n* = 6). Arrows indicated administration scheme. (C) Excised tumor weight (*n* = 6). (D) Change of average body weight after different treatments (*n* = 6). (E) Relative SDHA activity in tumor tissues (*n* = 4). Percentage of BCSCs as selected with marker CD44^+^CD24^−^ (F), side population (G), or CD133^+^ (H) in tumor tissues (*n* = 4). (I) Representative immunofluorescence staining images of Hif-1α and CD133 in tumor tissues. Scale bar: 100 μm. (J) Schematic illustration for the method to construct the subcutaneous BCSC tumor model. (K) Tumor growth curves (*n* = 6). Arrows indicated administration scheme. (L) Excised tumor weight (*n* = 6). (M) Quantification of metastatic pulmonary nodule area after different treatments (*n* = 3). (N) H&E staining for lung metastasis analysis after different treatments. Red arrows indicated the metastatic nodules. Data represent mean ± SEM, n.s., not significant; *P < 0.05; **P < 0.01; ***P < 0.001.

The in vivo phototherapeutic efficiency of the CyOA NPs was evaluated using a subcutaneous 4T1 tumor model (Fig. 5A). Hiporfin, an approved photosensitizer by the National Medical Products Administration of China, was employed as a positive control. As shown in Fig. 5B and C, compared to aggressive tumor growth in the saline group, mice that received CyOA NPs without irradiation showed a tumor inhibition rate (TIR) of 42%, which is slightly lower than that of Hiporfin (TIR of 53%). As expected, if the tumor sites were exposed to irradiation after intravenous injection of CyOA NPs, TIR was distinctly enhanced to 67%. The best antitumor efficacy in the CyOA NPs + L group was also evidenced by the smallest tumor in excised tumor images (Fig. S14), the most extensive necrosis in hematoxylin and eosin (H&E) staining, and the lowest proliferation Ki67 fluorescence staining (Fig. S15). Consistent with in vitro results, CyOA NPs significantly reduced intratumoral SDHA activity and alleviated intratumoral hypoxia (Fig. 5E and I). We further explored the potency of CyOA NPs to selectively suppress BCSCs in vivo after 14 days of treatment. As illustrated in Fig. 5F to H, compared to the other 3 groups, the lowest CSC ratio was always observed in the CyOA NPs + L group. In detail, compared to the saline group, CyOA NPs + L treatment reduced the proportion of CD133^+^ tumor cells by 24.5%, side population cells by 94.1%, and CD44^+^CD24^−^ tumor cells by 30.3%. Besides, CD133 fluorescence staining further demonstrated that CyOA NPs + L treatment could potently eradicate CSCs in subcutaneous 4T1 tumor tissues (Fig. 5I and Fig. S16).

### In vivo antitumor efficacy in the subcutaneous BCSC tumor model

To gain more insights into the merits of the multifunctional self-assembled hemicyanine–oleic acid conjugate, we further investigated the *in vivo* antitumor and anti-metastatic efficacy of CyOA NPs in the subcutaneous BCSC tumor model. As CSCs are highly tumorigenic, only 100,000 BCSCs, one-tenth of the subcutaneous 4T1 model, were injected subcutaneously into each mouse (Fig. 5J). The treatment schedule was consistent with the subcutaneous 4T1 tumor model. Tumor growth curves and excised tumor images were recorded for each mouse (Fig. S17). As shown in Fig. 5K and L, the highest tumor volume (73%) and weight (83%) inhibition rates further validated the best antitumor effect of CyOA NPs + L in the BCSC tumor model among all groups. Moreover, compared to 53% TIR in the 4T1 tumor model, Hiporfin + L showed a limited ability to suppress tumor growth in the BCSC tumor model, with a TIR of 33%. In contrast, the antitumor efficacy of CyOA NPs + L increased by almost 20% in the BCSC tumor model relative to the conventional 4T1 tumor model. These results further corroborated that alleviating tumor hypoxia and enabling Mito-PDT could solve inherent drawbacks of traditional PDT, especially for CSCs. Since CSCs have been broadly recognized to facilitate distant metastasis, we further investigated the area of metastatic lung nodules by H&E staining (Fig. 5N). Quantitative results showed that the CyOA NPs − L and Hiporfin + L group displayed marginal inhibition effects on metastasis, and the average metastatic area in these groups was up to almost 0.4 mm^2^ per lung (Fig. 5M). In contrast, CyOA NPs + L had almost no lung metastasis by day 22. These results confirmed the phototherapeutic effectiveness of CyOA NPs in eradicating CSCs, suppressing tumor growth, and inhibiting tumor metastasis.

### In vivo safety evaluation

The in vivo safety of intravenously administered CyOA NPs was systematically evaluated in 4T1 tumor-bearing mice. During the treatment period, mice in all groups remained healthy, with good appetite, shiny and soft fur, and slightly increased weight (Fig. 5D). In addition, there was no notable histopathological difference in major organs (heart, liver, spleen, kidney, and lung) after the last treatment (Fig. S18). Besides, both blood biochemistry [alanine aminotransferase (ALT), aspartate aminotransferase (AST), creatine kinase (CK), blood urea nitrogen (BUN), and creatinine (CREA)] and blood routine [white blood cell (WBC), red blood cell (RBC), platelet (PLT), and hemoglobin (HGB)] (Fig. S19) measurements were within normal ranges. These results confirmed that CyOA NPs-mediated Mito-PDT exhibited no indication of toxicity in vivo.

## Discussion

In summary, unlike traditional O_2_ economizers with multiple ingredients, we developed a versatile small-molecule, hemicyanine–oleic acid conjugate, to combat hypoxia resistance in PDT. This study has 3 advantages. First, we confirmed that the oleic acid modification promoted the self-assembly of hemicyanine–oleic acid conjugates in aqueous solution without any exogenous excipients, which enhanced photosensitizer accumulation in tumor tissues. Second, we demonstrated that CyOA NPs were able to reduce OCR by targeting SDHA, thereby conserving endogenous O_2_ for subsequent Mito-ROS burst. Third, we illustrated that O_2_ economical PDT, endowed by CyOA NPs, can dramatically enhance effects against BCSCs, leading to higher tumor inhibition and less lung metastasis in vivo, even compared to a clinically used photosensitizer (Hiporfin). This work provides an exquisite example, based on derivatization of commonly used dye, to solve inherent bottlenecks in PDT of cancer treatments.

We acknowledge some limitations of this study. First, NIR light at 660 nm is widely used in PDT, but its limited penetration depth prevents clinical applications; further modification of the structure of CyOA is warranted to redshift its absorption. Second, as CyOA is an all-in-one small molecule that integrates O_2_ conservation and Mito-ROS burst under light, the duration of administration and light treatment requires further cautious optimization. Third, ROS are double-edged swords in CSCs; only high ROS levels induce CSC death, while low to moderate ROS levels are favorable for CSC self-renewal and proliferations. Thus, the combination of PDT with other agents to disturb antioxidant systems is an ideal choice for synergistically eradicating CSCs.

## Materials and Methods

### Materials and general information

All reagents were purchased from commercial suppliers except otherwise stated. Marketed reagents were used with no further purification. IR780, IR783, resorcinol, oleic acid, *N*-ethyl-*N*'-(3-dimethylaminopropyl) carbodiimide hydrochloride (EDC), 4-dimethylaminopyridine (DMAP), TEMP, and DPBF were purchased from Aladdin Reagent Inc. (Shanghai, China). Lyso-Tracker Red, Mito-Tracker green, ATP Assay Kit, and Immunoprecipitation Kit with Protein A+G Magnetic Beads were purchased from Beyotime Biotechnology (Shanghai, China). DCFDA was obtained from MedChemExpress (Junction, NJ, USA). The Mitochondria Isolation Kit and the SDHA Activity Assay Kit were obtained from Solarbio Co., Ltd. (Beijing, China). MitoSOX Red and Cell Counting Kit-8 (CCK-8) were bought from Yeasen Biotech Co., Ltd. (Shanghai, China). Trypsin-EDTA (0.05%), FBS and RPMI1640 and DMEM/F12 cell culture media were purchased from Gibco Life Technologies. Brilliant Violet 421 anti-mouse CD133, FITC anti-mouse CD24, and PE anti-mouse/human CD44 were obtained from BioLegend. The ROS-ID Hypoxia/Oxidative Stress Detection Kit was purchased from Enzolifesciences. Column chromatography was conducted using silica gel (200 to 300 mesh). Ultraviolet/visible (UV/Vis) spectra were acquired on a Persee TU-1901 spectrophotometer. Fluorescence spectra were recorded by a spectrofluorometer (Fluoromax^+^, HORIBA, Japan). Nuclear magnetic resonance (NMR) spectra were measured on a Bruker ASCEND 600 MHz NMR magnet system and HRMS spectra were recorded by a SolariX 7.0T mass spectrometer (Bruker Daltonics, USA). The size and morphology of NPs were characterized by DLS (Malvern Zetasizer Nano-ZS, U.K.) and TEM (JEM-1230; JEOL, Japan). A CLSM (Olympus, FV3000) was used to capture the intracellular fluorescence. OCR was measured using a Clark oxygen electrode (Unisense, Denmark) and Seahorse XF96 MitoStress test (Agilent, Santa Clara, California, USA). Biodistribution of drugs was performed with an in vivo imaging system (IVIS Lumina XR, Caliper, USA)

### Synthesis of CyOH

CyOH was synthesized with reference to published literature [38]. Briefly, resorcinol (220 mg, 2 mmol) and K_2_CO_3_ (276 mg, 2 mmol) were stirred in anhydrous *N*,*N*-dimethylformamide (DMF) (5 ml) at room temperature for 30 min. Then, a solution of IR780 (667 mg, 1 mmol) in DMF (2 ml) was added to the above mixture and heated at 60 °C under N_2_ atmosphere for 5 h. Eventually, the solvent was evaporated under reduced pressure, and the residue was purified by column chromatography on silica gel (MeOH/CH_2_Cl_2_ = 1:20 →1:10) to afford the dark blue solid (Yield 68%). The compound SO_3_-CyOH was synthesized using the same method as CyOH.

### Synthesis of CyOA

A mix of oleic acid (282 mg, 1 mmol), EDC (192 mg, 1 mmol), and DMAP (24.3 mg, 0.2 mmol) in 10 ml of CH_2_Cl_2_ was added in a round-bottom flask. Then, a solution of CyOH (431 mg, 0.8 mmol) in CH_2_Cl_2_ (5 ml) was added to the above mixture and stirring was continued at room temperature under a N_2_ atmosphere for 8 h. After the reaction was completed, the mixture was extracted and the organic phase was collected and concentrated under vacuum, and the residue was purified by column chromatography on silica gel (MeOH/CH_2_Cl_2_ = 1:20 →1:10) to afford the dark blue oil (Yield 71%). ^1^H NMR (600 MHz, CDCl_3_) δ 8.62 (d, *J* = 15.0 Hz, 1H), 7.52–7.48 (m, 3H), 7.44–7.36 (m, 2H), 7.11 (s, 1H), 7.07–7.03 (m, 1H), 7.00–6.96 (m, 1H), 6.87 (d, *J* = 15.0 Hz, 1H), 5.37–5.32 (m, 2H), 4.75–4.61 (m, 2H), 2.88–2.82 (m, 2H), 2.76–2.70 (m, 2H), 2.61 (t, *J* = 7.6 Hz, 2H), 2.10–1.92 (m, 10H), 1.79 (s, 6H), 1.31–1.22 (m, 21H), 1.12–1.06 (m, 3H), 0.88–0.85 (m, 3H). ^13^C NMR (151 MHz, CDCl_3_) δ 178.8, 171.9, 159.8, 153.2, 152.8, 146.4, 142.3, 141.6, 130.9, 130.2, 129.8, 129.5, 128.2, 128.0, 122.5, 119.8, 119.1, 115.8, 113.7, 109.5, 107.0, 51.1, 48.0, 34.5, 32.0, 29.9, 29.8, 29.6, 29.5, 29.4, 29.3, 29.2, 28.30, 27.4, 27.3, 24.9, 24.6, 22.8, 21.8, 21.5, 20.4, 14.3, 11.7. HRMS (ESI): *m/z* calcd for C_46_H_62_NO_3_^+^ [M]^+^ 676.4724, found 676.47155.

### Synthesis of SO_3_-CyOA

The compound SO_3_-CyOA was synthesized using the same method as CyOA (Yield 64%). ^1^H NMR (600 MHz, CDCl_3_) δ 8.59 (d, *J* = 15.0 Hz, 1H), 7.54–7.47 (m, 2H), 7.46–7.42 (m, 1H), 7.41–7.31 (m, 1H), 7.36–7.31 (m, 1H), 7.07–6.88 (m, 4H), 5.42–5.26 (m, 2H), 4.59–4.49 (m, 2H), 3.11–2.99 (m, 2H), 2.88–2.77 (m, 2H), 2.69–2.56 (m, 4H), 2.19–2.08 (m, 4H), 2.05–1.97 (m, 4H), 1.92–1.86 (m, 2H), 1.75 (s, 8H), 1.35–1.22 (m, 20H), 0.86 (t, *J* = 6.9 Hz, 3H). ^13^C NMR (151 MHz, CDCl_3_) δ 178.4, 171.9, 159.8, 153.1, 152.7, 146.8, 142.2, 141.3, 130.7, 130.2, 129.8, 129.7, 128.2, 127.9, 122.4, 119.8, 119.0, 116.5,113.6, 109.4, 107.0, 50.9, 49.8, 46.3, 34.5, 32.0, 29.9, 29.8, 29.7, 29.6, 29.4, 29.3, 29.2, 29.2, 28.3, 27.4, 27.3, 27.3, 26.5, 24.9, 24.3, 22.8, 22.8, 20.4, 14.3. HRMS (ESI): *m/z* calcd for C_47_H_63_NO_6_S [M+H]^+^ 770.4449, found 770.44373.

### Spectroscopic data

For UV/Vis measurements, compounds CyOH, SO_3_-CyOH, CyOA, and SO_3_-CyOA were dissolved in dimethyl sulfoxide (DMSO) at 50 μM. For fluorescence spectroscopic studies, CyOA and SO_3_-CyOA were dissolved in saline or saline containing porcine liver esterase (PLE, 100 U/ml) to make the stock solutions (500 μM), which were then diluted to 0.5 μM with DMSO at the designed time intervals as the testing solutions.

### Preparation and characterization of NPs

The self-assembly of CyOA NPs and SO_3_-CyOA NPs occurred spontaneously. Briefly, 200 μl of 10 mM CyOA or SO_3_-CyOA dissolved in methanol was added to 4 ml of deionized water. After evaporation of organic solvent, the NP was obtained as a blue solution without visible precipitation. The particle size, polydispersity index, and zeta potential of NPs were measured by DLS. The morphology of NPs was characterized by TEM.

### Measurement of ^1^O_2_ generation in solution

We adopted 2 methods to measure ^1^O_2_ generation in solution. The first method aims to monitor the photooxidation of DPBF. Concretely, 1 mg of DPBF was dissolved in 1 ml of ethanol to obtain the stock solution. Then, 120 μl of DPBF solution was added into 3,880 μl of 2.5 μM CyOH, SO_3_-CyOH, CyOA NPs, and SO_3_-CyOA NPs solutions, respectively. The mixture was exposed at 660 nm laser irradiation (200 mW/cm^2^). At the designed time points, 100 μl of the above mixture was taken out and mixed with 100 μl of ethanol, and then the absorbance of the mixture at 406 nm was recorded. EPR spectroscopy was also used to further confirm ^1^O_2_ production of photosensitizers under 660 nm laser irradiation. Typically, 20 μl of TEMP was first added into 1 ml of CyOA NPs or SO_3_-CyOA NPs solution (10 μM). The mixture was then exposed at 660 nm laser irradiation (200 mW/cm^2^) for 120 s. Immediately afterwards, the ^1^O_2_ signal was detected by the EPR spectrometer (Bruker EMXmicro-6/1/P/L). Moreover, no light exposure to CyOA NPs or SO_3_-CyOA NPs was also tested for comparison.

### Cell culturing and cytotoxicity test for adherent 4T1 cells

4T1 cells were cultured in RPMI1640 medium supplemented with 10% FBS, 100 units/ml penicillin, and 100 μg/ml streptomycin in a 37 °C humidified atmosphere containing 5% CO_2_. To construct the in vitro hypoxic tumor models, 4T1 cells were transformed into a culture chamber with a humidified hypoxic atmosphere (1% O_2_, 5% CO_2_, and 94% N_2_) at 37 °C and incubated for more than 12 h. To investigate the phototoxicity of CyOA NPs and SO_3_-CyOA NPs, 4T1 cells were seeded in 96-well cell culture plates at a density of 10,000 cells per well for 12 h. CyOA NPs and SO_3_-CyOA NPs at different concentrations were added into each well and incubated for 4 h. The cells were replaced with fresh medium and then irradiated upon 660 nm laser at 200 mW/cm^2^ for 3 min. Cell viability was determined with CCK-8. Moreover, dark toxicity of CyOA NPs was also assayed.

### Breast cancer stem cells

Mammospheres formation test in suspension is an established model for culturing CSCs [4]. In particular, single 4T1 cells were prepared using trypsin, and then plated in ultra-low attachment culture dishes at a density of 10,000 cells/cm^2^ with mammosphere medium (DMEM/F12, B-27, 10 ng/ml bFGF, 20 ng/ml hEGF, 0.4% low endotoxin bovine serum albumin, and 4 μg/ml insulin). After 5 days for culture, mammospheres were collected for the next cytotoxicity assay.

### Tumor-repopulating cells

Culture of tumor cells in 3D fibrin gels was performed as previously reported [37]. Briefly, 4T1 cells were without any pretreatment or pretreatment with either SO_3_-CyOA NPs (20 μM) or CyOA NPs (2 μM) for 4 h, and then cells were irradiated with laser for 3 min (660 nm, 200 mW/cm^2^). Light-treated cells (4×10^4^ cells/ml) were mixed with fibrinogen (2 mg/ml) at a 1:1 ratio, then 50-μl mixtures were seeded into 96-well plates and blended with preloaded 1 μl of thrombin (0.1 U/μl). After 20 min, 200 μl of RPMI1640 medium with 10% FBS was supplemented. The size and number of colonies were measured or counted in 15 randomly chosen regions.

### Confocal fluorescence imaging

CLSM imaging was performed to track intracellular localization of photosensitizers and intracellular hypoxia level of 4T1 cells after treatment with SO_3_-CyOA NPs or CyOA NPs. For co-localization analysis, cells were seeded in a 20-mm confocal dish. After 12 h, the medium was changed with fresh medium containing SO_3_-CyOA NPs and CyOA NPs, respectively. Then, after incubation at 37 °C for 8 h, the cells were stained with Lyso-Tracker and Mito-Tracker, and then imaged with CLSM. The intracellular hypoxia level was confirmed by hypoxia/oxidative stress detection kit ROS-ID. Briefly, 4T1 cells were seeded on a confocal dish for 12 h, then treated with SO_3_-CyOA NPs (20 μM) or CyOA NPs (2 μM) for 4 h. After washing 3 times with PBS, the cells were stained with a hypoxia probe according to the manufacturer’s protocol, and then imaged by CLSM. Moreover, intracellular hypoxia level was also evaluated by flow cytometry analysis with the same treatment scheme.

### Measurement of mitochondrial uptake of NPs

Mitochondrial uptake of SO_3_-CyOA NPs and CyOA NPs was quantified by measuring their fluorescence intensity. 4T1 cells were seeded in 100-mm dishes and treated with SO_3_-CyOA NPs (20 μM) or CyOA NPs (2 μM) for 6 h. Mitochondrial isolation was then performed using the cell mitochondrial isolation kit. After centrifugation, the bottom mitochondrial pellets were diluted in 100 μl of PBS and the mixtures were used for the determination of SO_3_-CyOA and CyOA fluorescence by a multimode microplate reader. The supernatants were used for the quantification of SO_3_-CyOA and CyOA in the cytoplasm.

### OCR test

We adopted 2 methods to measure OCR. The first method is to monitor the dissolved oxygen with a Clark oxygen electrode (Unisense, Denmark). A total of 1×10^6^ 4T1 cells were seeded in 6-well plates and treated with SO_3_-CyOA NPs (20 μM) or CyOA NPs (2 μM) for 6 h, and then resuspended in 1 ml of Hanks’ Balanced Salt Solution. The medium was sealed with liquid paraffin to avoid oxygen exchange. Dissolved oxygen was recorded every 1 s for a total of 2 min. The second method is to detect OXPHOS using Seahorse XFe96 MitoStress Test. 4T1 cells were seeded on a Seahorse XFe96 well plate at 3,000 cells/well and incubated overnight (37 °C, 5% CO_2_). Cells were then treated with SO_3_-CyOA NPs (20 μM) or CyOA NPs (2 μM) for further 6 h. Cells were washed and changed to XF basal medium 1 h before the experiment. Port injections were prepared in the same media and loaded into the cartridge with port A containing oligomycin (1.5 μM post-injection), port B containing trifluoromethoxy carbonylcyanide phenylhydrazone (FCCP) (1 μM post-injection), and port C containing rotenone and antimycin A (0.5 μM each post-injection). For all analysis, values were normalized to cell number. The fold change was calculated relative to the absence SO_3_-CyOA NPs and CyOA NPs.

### Detection of intracellular ATP

4T1 cells (2×10^5^ cells) were incubated with SO_3_-CyOA NPs (20 μM) or CyOA NPs (2 μM) for 12 h in a 6-well plate. Intracellular ATP levels were tested according to the Enhanced ATP Assay Kit instructions.

### Western blot assay

4T1 cells were cultured in a hypoxic environment (1% O_2_, 5% CO_2_, and 94% N_2_) to assess intracellular Hif-1α levels. After 20 μM SO_3_-CyOA NPs or 2 μM CyOA NPs treatment for 12 h, cells were harvested. The protein content of cell lysate was quantified with a BCA Protein Quantification Kit. Samples were separated by 10% SDS-PAGE, blocked with 5% bovine serum albumin, then incubated with primary antibody to Hif-1α (1:1,000, Abcam, ab179483) and subsequently peroxidase-conjugated secondary antibody. The level of Hif-1α was visualized using a chemiluminescent detection system (Clinx Science Instruments Co., Ltd., China).

### Molecular docking

The crystallographic structure of SDH from *G. gallus* (PDB code: 2FBW) was accessed from the RCSB PDB database. With genetic algorithm and MMFF94 force field, Openbabel 2.4.1 was employed to convert the structure of CyOH and CyOA from 2 to 3 dimensions [39]. Docking analysis was performed using AutoDock Vina [40]. Search grid was identified as center x: 10.177, center y: 16.429, and center z: 8.67, with dimensions of 30 in all directions. The lowest binding energy pose was selected and visually analyzed using PyMOL software (Version 2.4.0a0, Schrödinger, LLC).

### Pull-down experiment assay

4T1 cells were incubated with CyOA NPs (2 μM) for 4 h. After washing with PBS, cells were lysed and centrifuged; 30 μl of the supernatant was applied as the input. For immunoprecipitation (IP), cell lysates were incubated with SDHA antibody pre-coated magnetic beads following the protocol of Immunoprecipitation Kit with Protein A+G Magnetic Beads, and immunoglobulin G (IgG) antibody was used as a control. After the protein complex was centrifuged and washed, it was separated by 10% SDS–PAGE and transferred to a polyvinylidene fluoride membrane. After blocking, the membrane was co-incubated with SDHA antibody and, subsequently, peroxidase-conjugated secondary antibody. The fluorescence signal was imaged using an Odyssey CLx infrared imaging system (LI-COR Bioscience, USA). The SDHA signal was visualized using a chemiluminescent detection system.

### Measurement of intracellular SDHA activity

4T1 cells (5 × 10^6^ cells) were incubated with SO_3_-CyOA NPs (20 μM) or CyOA NPs (2 μM) for 12 h in 25-cm^2^ cell culture flasks. Intracellular SDHA activity was measured by the SDHA Activity Assay Kit (Solarbio Co., Ltd., China).

### Intracellular ROS generation detection

Intracellular total ROS under light irradiation was measured using the fluorescent probe DCFDA, while Mito-ROS was detected using Mito-SOX [41]. Cells were cultured in 6-well plates for 12 h, then the medium was replaced with PBS, SO_3_-CyOA NPs (20 μM) or CyOA NPs (2 μM) for 4 h, followed by adding DCFDA (10 μM) or Mito-ROS (5 μM) for another 30 min. Afterward, cells were washed with fresh PBS buffer and irradiated upon 660 nm laser at 200 mW/cm^2^. Intracellular fluorescence intensity of DCF and SOX was quantified by flow cytometry. Moreover, intracellular ROS under hypoxic conditions were measured using cells cultured in hypoxic environment and hypoxic medium.

### Transwell migration assay

After co-incubation with PBS, SO_3_-CyOA NPs (20 μM), or CyOA NPs (2 μM) for 4 h, 4T1 cells were replaced with fresh medium and then irradiated upon 660 nm laser at 200 mW/cm^2^ for 3 min. Afterward, 4T1 cells (1×10^4^) were suspended in serum-free medium and seeded into the upper chamber. The lower chambers were filled with 600 μl of RPMI1640 medium containing 10% FBS. The lower-layer cells were fixed with 4% paraformaldehyde solution after 24 h of incubation and then stained with crystal violet. Migrating cells were counted in 5 randomly selected areas.

### In vivo imaging

CyOH (5 μmol/kg) or CyOA NPs (5 μmol/kg) was injected intravenously into tumor-bearing mice. Fluorescence imaging was taken at different post-injection times (0, 30 min, 1 h, 2 h, 4 h, 8 h, 12 h, and 24 h). After 24 h post-injection, the mice were sacrificed, and the tumor tissues and major organs were taken for ex vivo imaging. All animal investigations were conducted following internationally accepted principles and Huazhong University of Science and Technology (HUST) guidelines for the care and use of laboratory animals.

### Pharmacokinetic studies

To explore whether unsaturated aliphatic chain modification could improve the pharmacokinetic properties of hemicyanine, we examined various pharmacokinetic parameters of CyOH and CyOA NPs. In detail, CyOH (5 μmol/kg) or CyOA NPs (5 μmol/kg) was injected intravenously into healthy mice. At pre-determined time points (5 min, 15 min, 30 min, 1 h, 2 h, 4 h, 8 h, 12 h, and 24 h), the plasma samples (30 μl) were collected and mixed with 90 μl of methanol. After centrifugation (8,000 *g*) to collect supernatants (100 μl), we measured photosensitizers’ concentration by their fluorescence intensity using a multimode microplate reader (Molecular Devices, Flex Station 3).

### Subcutaneous 4T1 tumor model

4T1 cells (1×10^6^) in 100 μl of PBS were injected into the right flank of female BALB/c mice. When tumor volume grew to about 50 mm^3^, mice were assigned randomly to 4 groups: (a) saline, (b) CyOA NPs − L, (c) CyOA NPs + L, and (d) Hiporfin + L. CyOA NPs or Hiporfin at 10 μmol/kg was injected i.v. once every 3 days for 4 injections, and PDT (660 nm, 200 mW/cm^2^, 5 min) was performed 12 h after injection. The size of the tumor was recorded every other day with Vernier calipers. Tumor volume (*V*) (mm^3^) = *L* × *W*^2^/2 (*L* and *W* represent the longest tumor length and the width of tumors, respectively). After 12 days, mice were euthanized and the tumors were collected, photographed, and weighted. Excised tumors were also used to quantify the proportion of CSCs and the level of Hif-1α.

### Subcutaneous BCSC tumor model

As BCSCs exhibit extremely high tumorigenicity, only 1×10^5^ BCSCs were injected subcutaneously into the right flank of female BALB/c mice. Grouping and treatment regimen were the same as those for the subcutaneous tumor model. Lung metastasis was examined using H&E staining on day 22.

### Flow cytometry analysis

To evaluate CSC ratio among 4T1 cells after different treatments, 4T1 cells were seeded in 12-well cell culture plates for 12 h, and then cultured with PBS, SO_3_-CyOA NPs (20 μM), or CyOA NPs (2 μM) for 4 h. Afterwards, cells were replaced with fresh medium and then irradiated using a 660-nm laser at 200 mW/cm^2^ for 3 min. After 12 h, cells were washed 3 times with PBS, and then stained with Brilliant Violet 421 anti-mouse CD133, FITC anti-mouse CD24, and PE anti-mouse/human CD44. CSC proportion: CD133^+^ or CD44^+^CD24^−^ population cells. To evaluate CSC ratio in vivo, tumors were harvested following final treatment, then clipped, digested, and filtered through a 70-μm cell strainer. Collected single-cell suspensions were then co-incubated with different fluorochrome-conjugated antibodies (including CD133, CD44, and CD24), and the gating strategy for flow cytometry analysis was the same as above. Hoechst 33342 was applied for side population cell analysis, and the gating strategy was the same as previously reported [42].

### Immunofluorescence staining and imaging

After 12 days of treatments, the excised tumor tissues were fixed in paraformaldehyde solution and embedded in paraffin, and then were sliced at 10-μm thickness for Hif-1α, CD133, and Ki67 staining, respectively. These immunofluorescence staining experiments were performed by Biossci Biotechnology Co., Ltd. (http://www.biossci.com/). The intensity of the fluorescence was quantified with ImageJ software.

### Safety evaluation

After 12 days of treatment, 4T1 tumor-bearing mice were euthanized. The major organs (including heart, liver, spleen, lung, and kidney) were extracted for H&E staining, and the blood samples were gathered for blood biochemistry analysis (ALT, AST, CK, BUN, and CREA) and blood routine test (WBC, HGB, RBC, and PLT).

### Statistical analysis

The results of all experiments were expressed as mean ± SEM and statistical differences between 2 groups were analyzed by a 2-tailed unpaired Student’s *t* test. n.s., not significance, *P < 0.05; **P < 0.01; ***P < 0.001; ****P < 0.0001. All statistical calculations were carried out using GraphPad Prism software.

## Data Availability

All data obtained or analyzed in this research are included in the manuscript and Supplementary Materials.

## Supplementary Materials

Supplementary 1 Scheme S1 Figs. S1 to S19 Tables S1 and S2
