# Supplementary material for: Unimolecular Self-Assembled Hemicyanine–Oleic Acid Conjugate Acts as a Novel Succinate Dehydrogenase Inhibitor to Amplify Photodynamic Therapy and Eliminate Cancer Stem Cells
Source: Research (Wash D C). 2023 Sep 6;6:0223. doi: 10.34133/research.0223 (PMC10482163; doi:10.34133/research.0223)
Supplement: Supplementary 1 — Scheme S1 Figs. S1 to S19 Tables S1 and S2 [file research.0223.f1.docx]

Supporting Information

**Unimolecular self-assembled hemicyanine-oleic acid conjugate acts as a novel succinate dehydrogenase inhibitor to amplify photodynamic therapy and eliminate cancer stem cells**

*Qiang Wang, Tian Yang, Shiyou Li, Chen Xu, Chong Wang, Yuxuan Xiong, Xing Wang, Jiangling Wan, Xiangliang Yang^*^ and Zifu Li ^*^*

**Scheme S1.** Synthesis of CyOA and SO_3_-CyOA. Reagents and conditions: (i) Resorcinol, K_2_CO_3_, DMF, 60 ^0^C, 5 h; (ii) Oleic acid, EDC, DMAP, CH_2_Cl_2_, rt, 8 h.


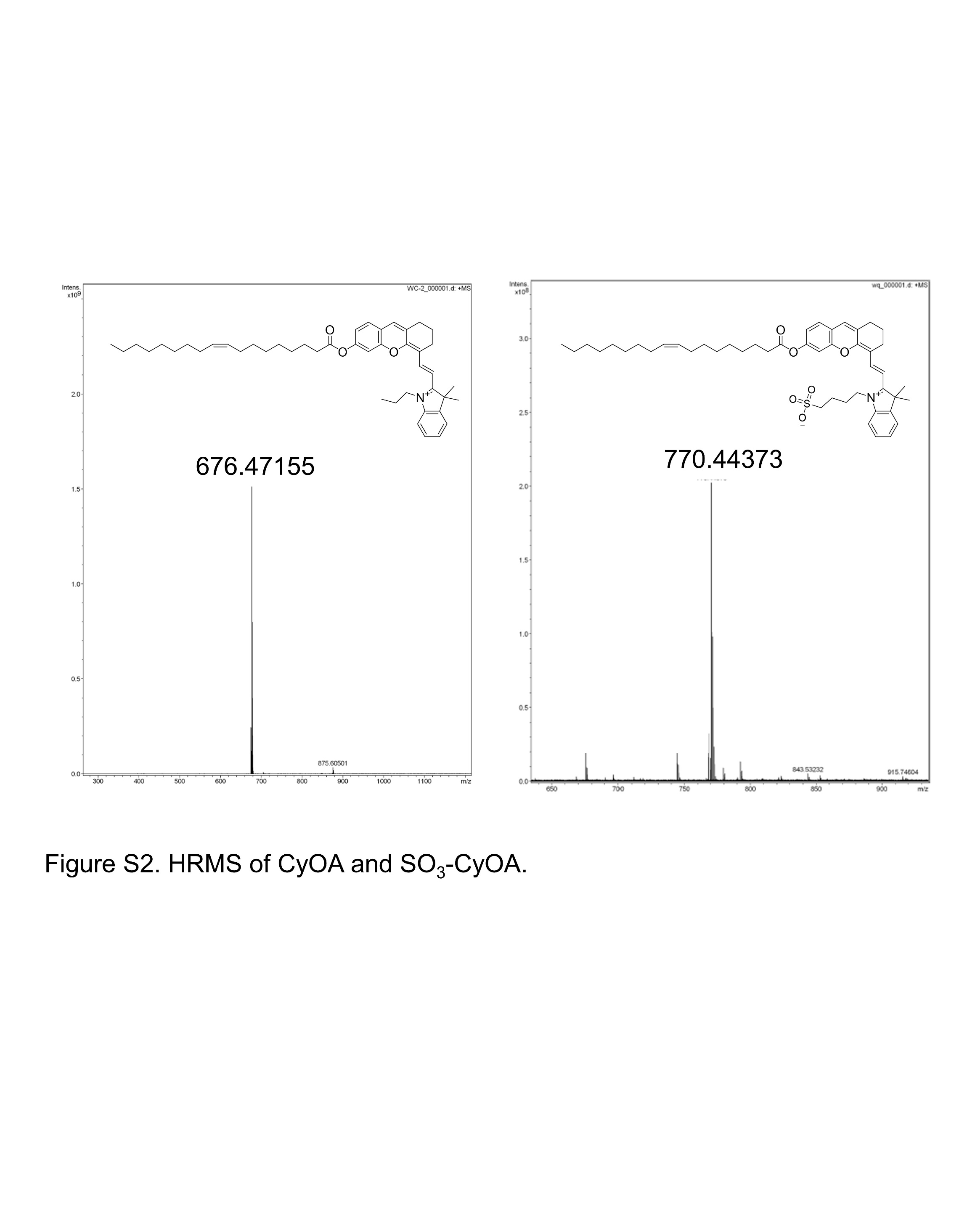


Figure S1. High-resolution mass spectra of CyOA (left) and SO_3_-CyOA (right).

**Figure S2.** ^1^HNMR and ^13^CNMR of CyOA.

**Figure S3.** ^1^HNMR and ^13^CNMR of SO_3_-CyOA.

**
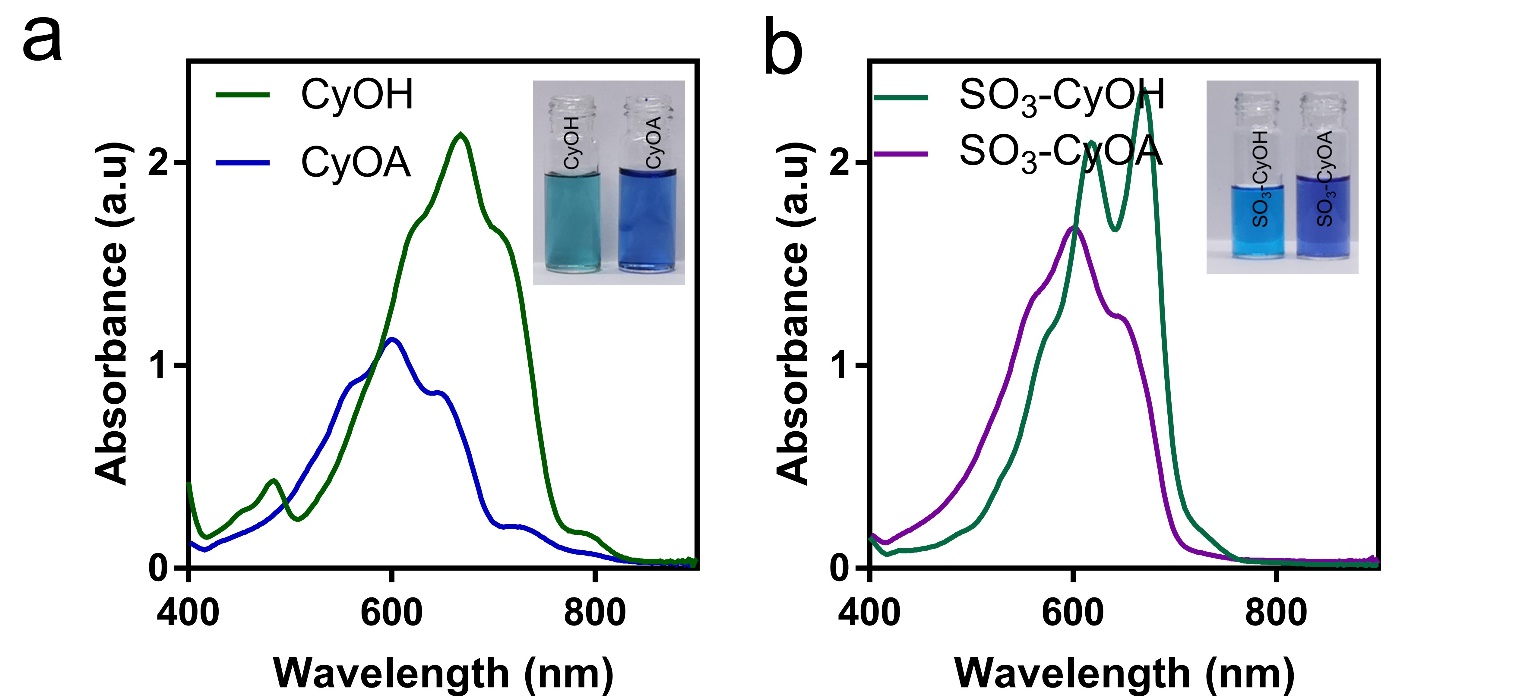
**

**Figure S4.** UV/Vis absorption of CyOA (a) and SO_3_-CyOA (b) in DMSO. Inset shows the images.


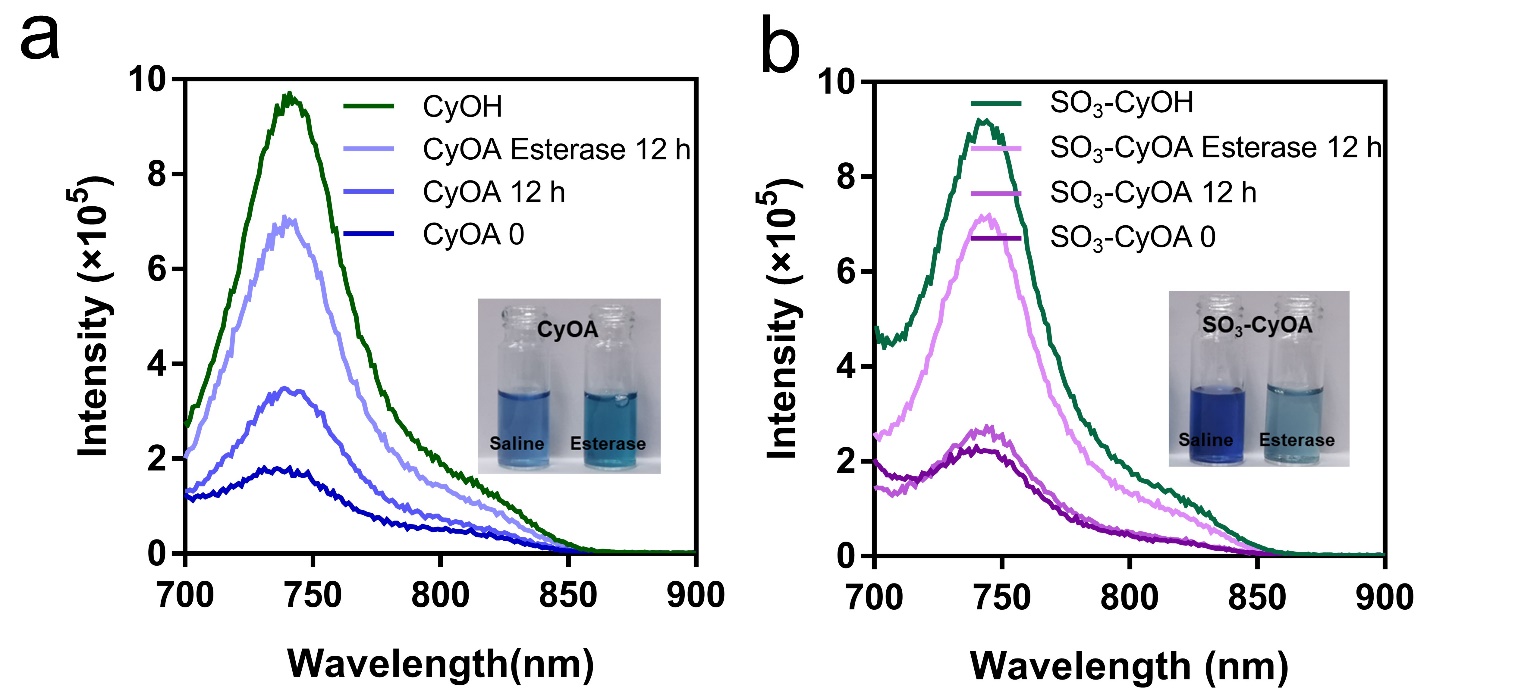


**Figure S5.** Fluorescence spectrum of CyOA (a) and SO_3_-CyOA (b) after co-incubation with esterase. Inset shows the images.


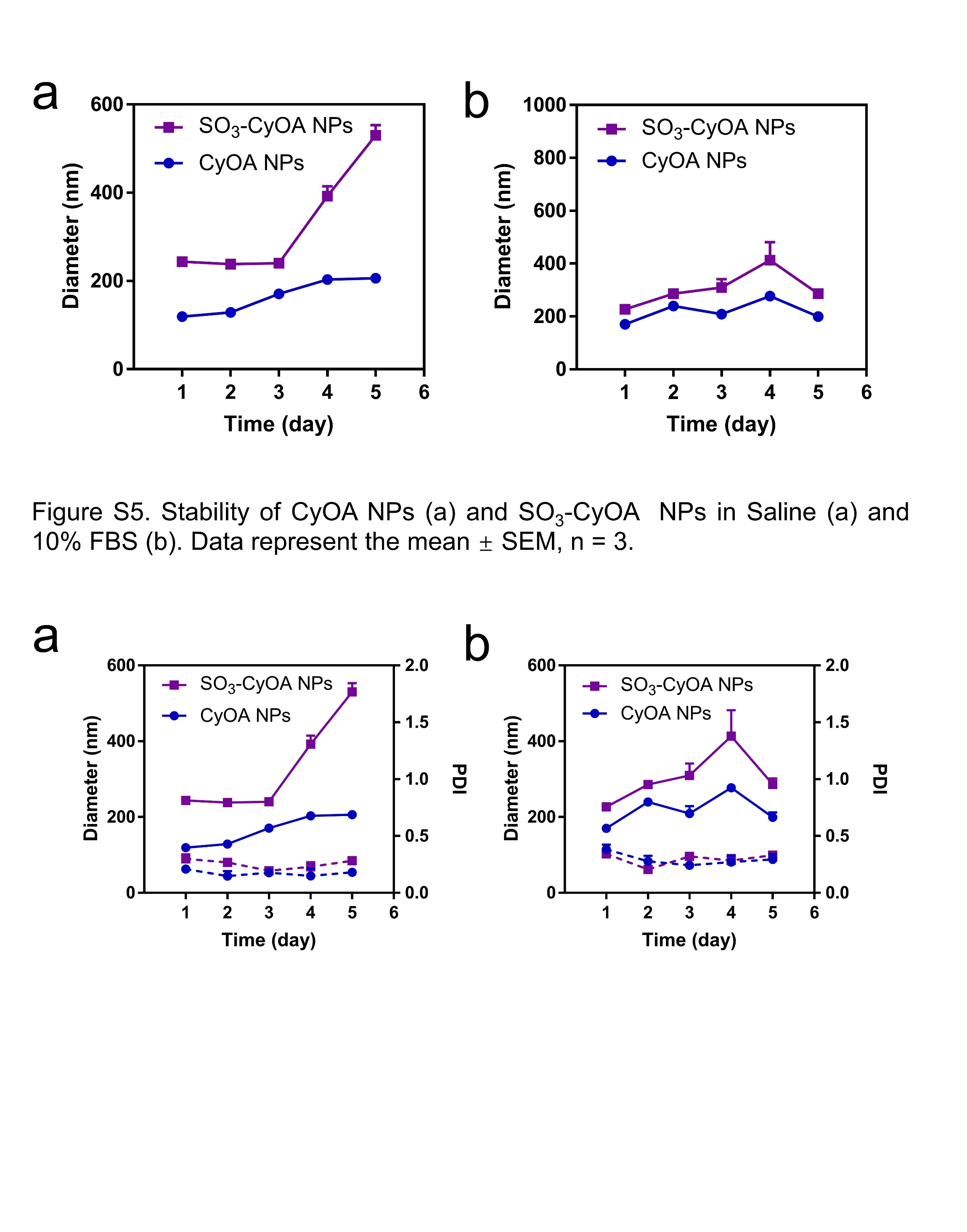


**Figure S6.** Stability of CyOA NPs (a) and SO_3_-CyOA NPs in saline (a) and 10% FBS (b). Data represent the mean ± SEM, n = 3.

**
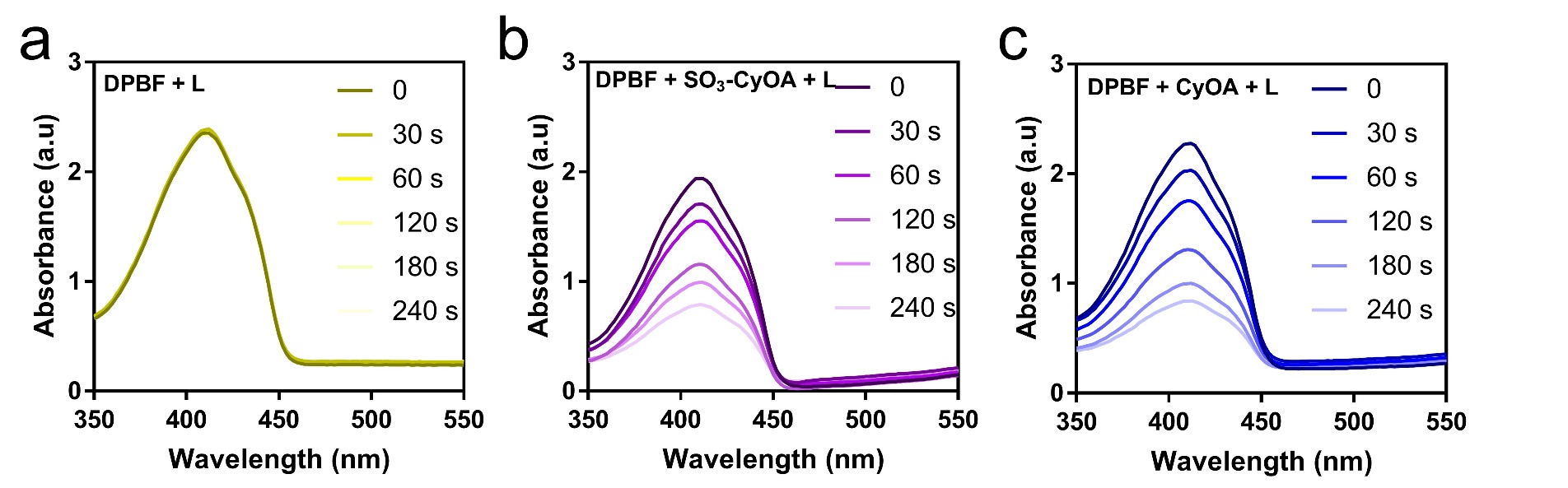
**

**Figure S7.** Photodegradation curves of DPBF (a) or treated with SO_3_-CyOA (b) or with CyOA (c) under 660 nm irradiation.

**
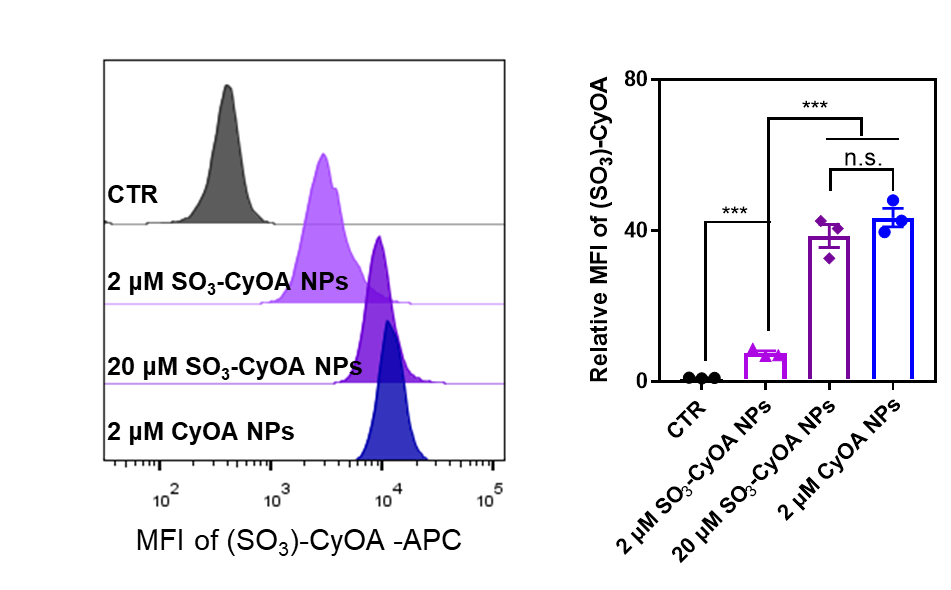
**

**Figure S8.** Cellular uptake of SO_3_-CyOA NPs or CyOA NPs as measured by flow cytometer. Data represent the mean ± SEM, n = 3; statistical significance was calculated by t-test; n.s., not significance, ***, p<0.001

**
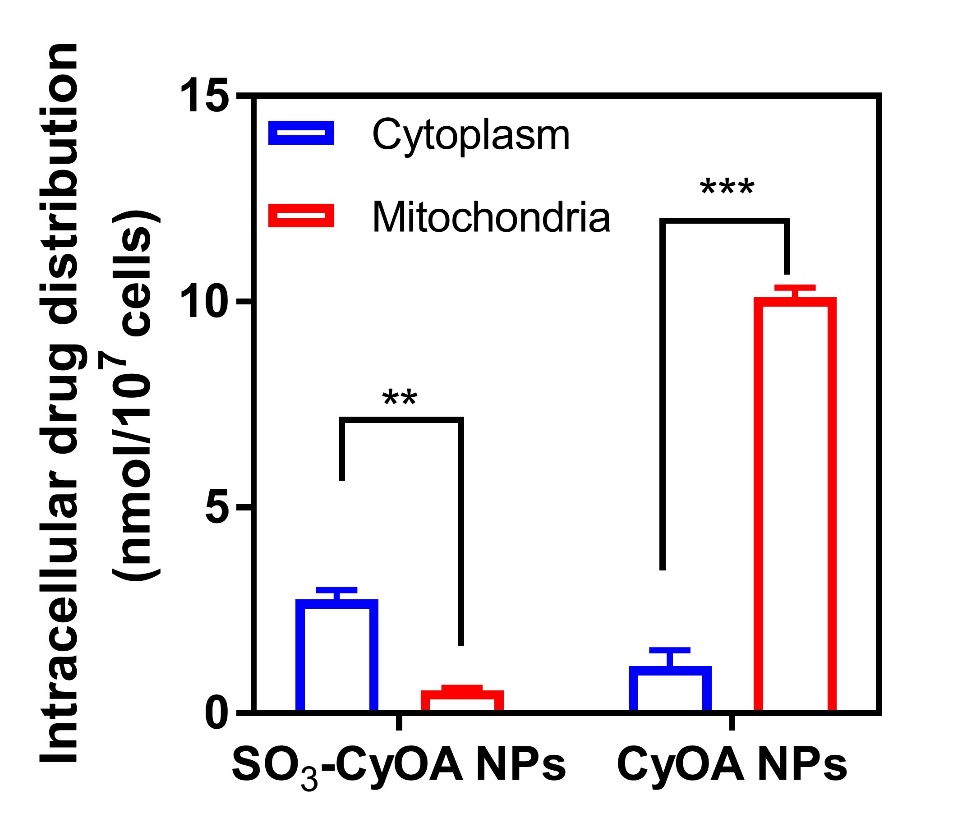
**

**Figure S9.** Quantification of CyOA and SO_3_-CyOA in different subcellular organelles. Mitochondria were isolated by differential centrifugation (n=3). Data represent mean ± SEM, statistical significance was calculated by t-test, **, *p*<0.01; ***, *p*<0.001.

**
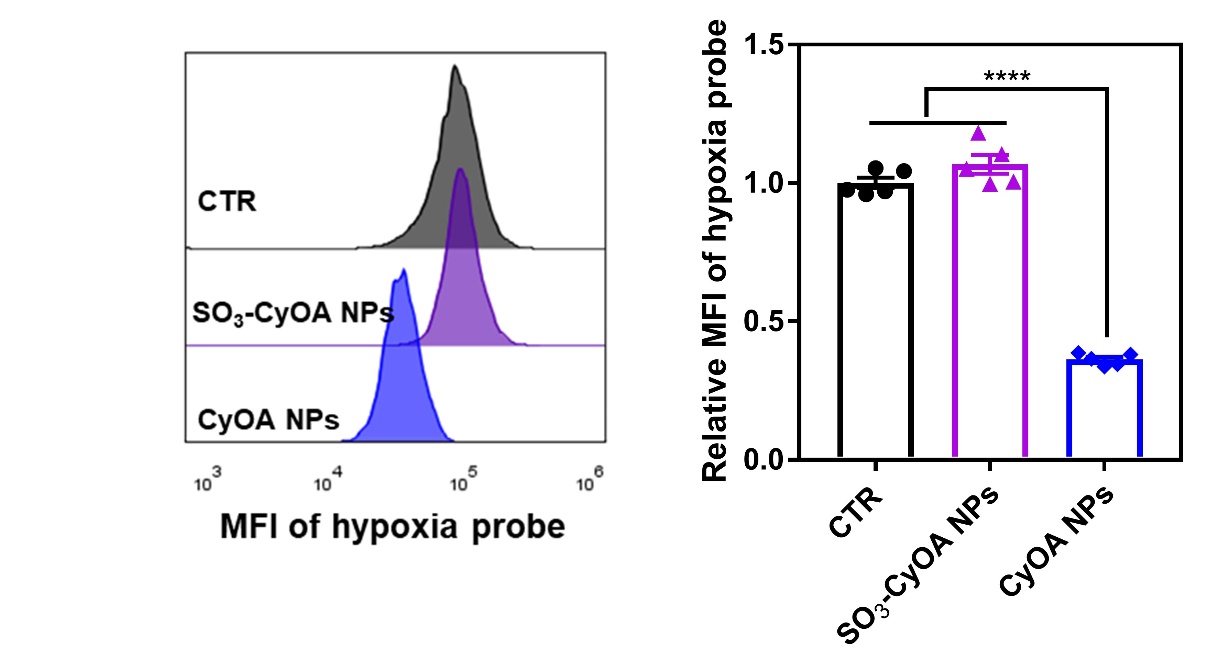
**

**Figure S10.** CyOA NPs alleviate intracellular hypoxia level as measured by flow cytometer. Data represent the mean ± SEM, n = 5; statistical significance was calculated by t-test; ****, *p<0.0001*.

**
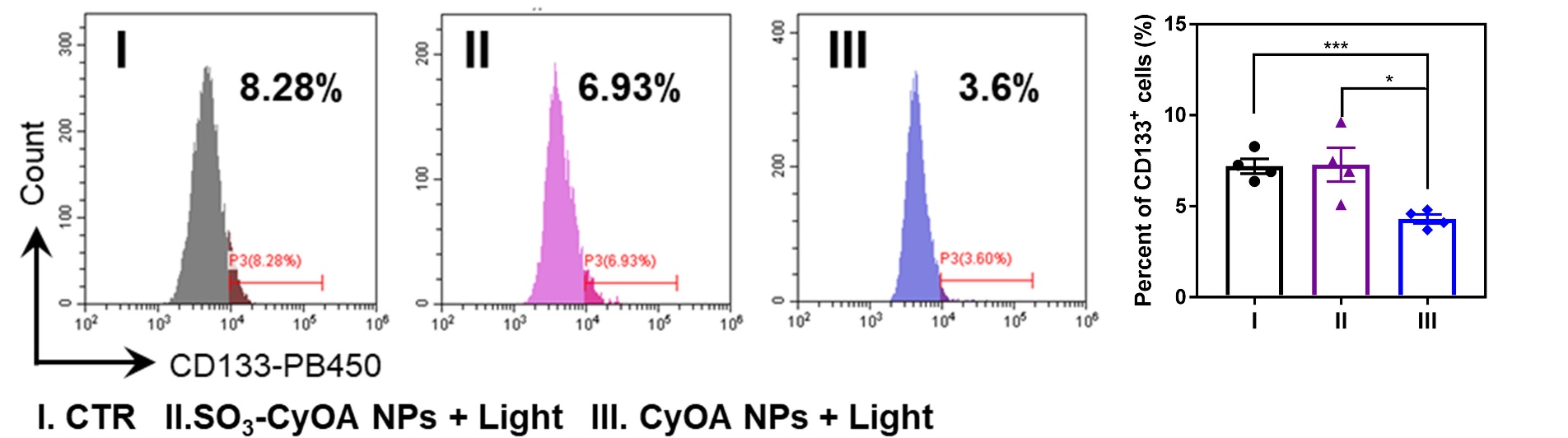
**

**Figure S11.** CyOA NPs diminish CD133^+^ population cell as measured by flow cytometer. Data represent the mean ± SEM, n = 4; statistical significance was calculated by t-test; *, *p<0.05*, ***, *p<0.001*.

**
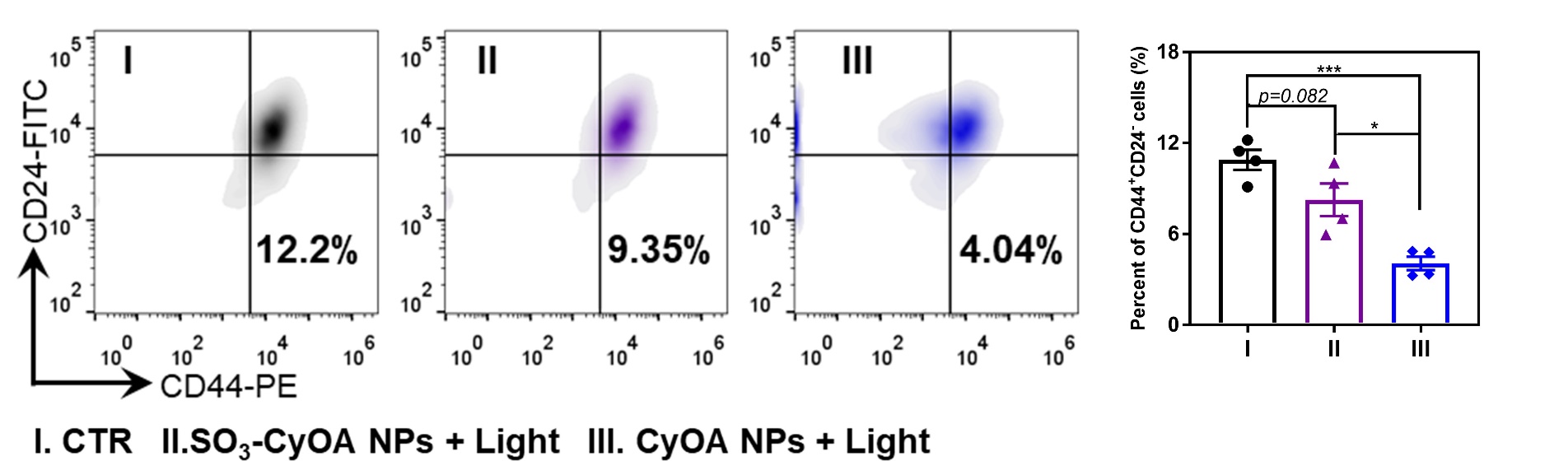
**

**Figure S12.** CyOA NPs diminish CD44^+^CD24^-^ population cell as measured by flow cytometer. Data represent the mean ± SEM, n = 4; statistical significance was calculated by t-test; *, *p<0.05*, ***, *p<0.001*.

**
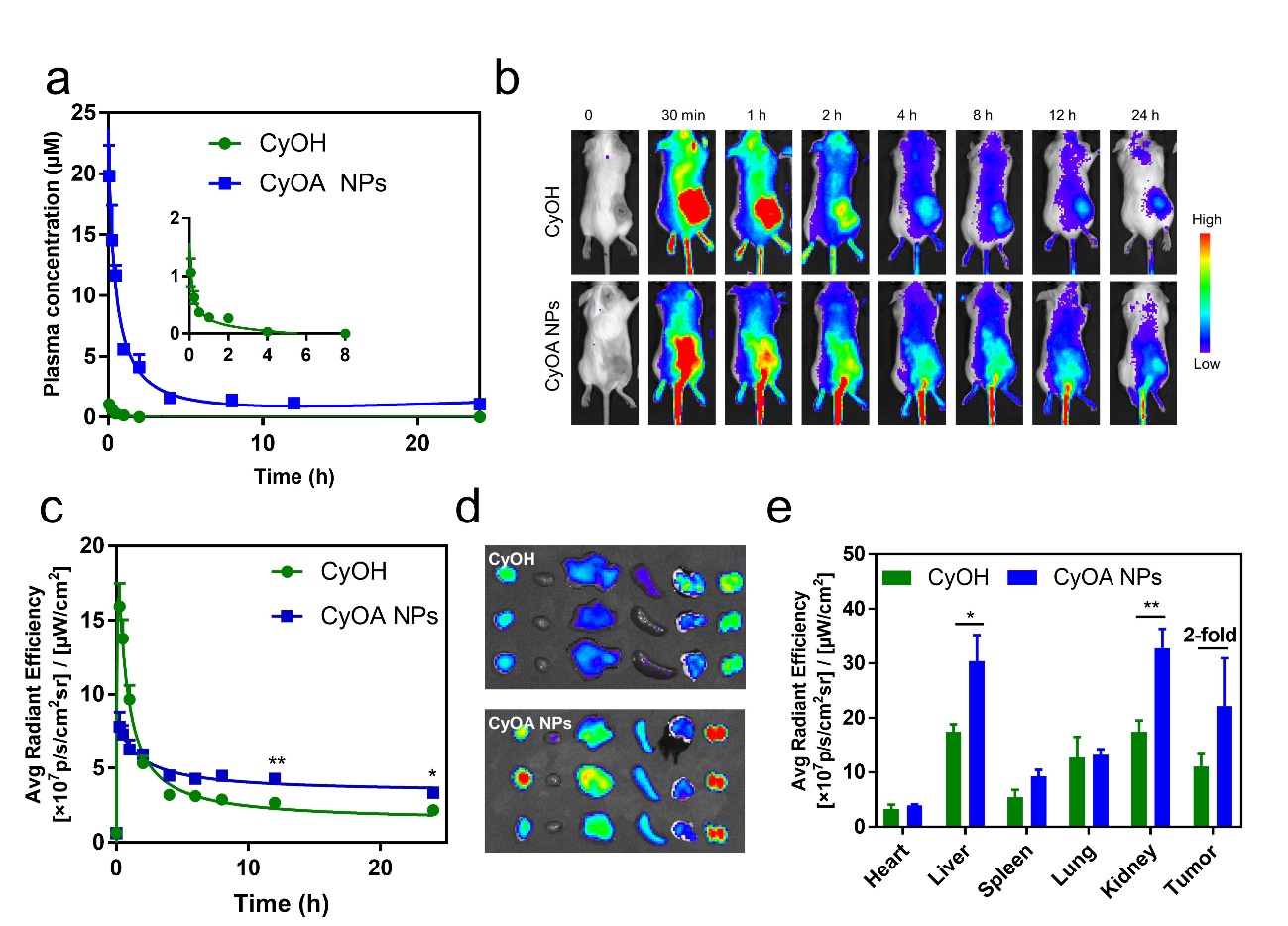
**

**Figure S13.** Unsaturated aliphatic chain-hemicyanine conjugates show longer half-life and higher tumor accumulation. a. Plasma concentration-time curves of CyOH and CyOA NPs. b. *In vivo* fluorescent images of 4T1 tumor bearing mice. c. Quantification of fluorescent intensity of tumors at predetermined time points. d. *Ex vivo* fluorescent images and quantification of fluorescent intensity of major organs and tumors 48 h after mice were i.v. injected with CyOH or CyOA NPs (e). Data represent the mean ± SEM, n = 3; statistical significance was calculated by t-test; *, *p<0.05*, **, *p<0.01*.

**
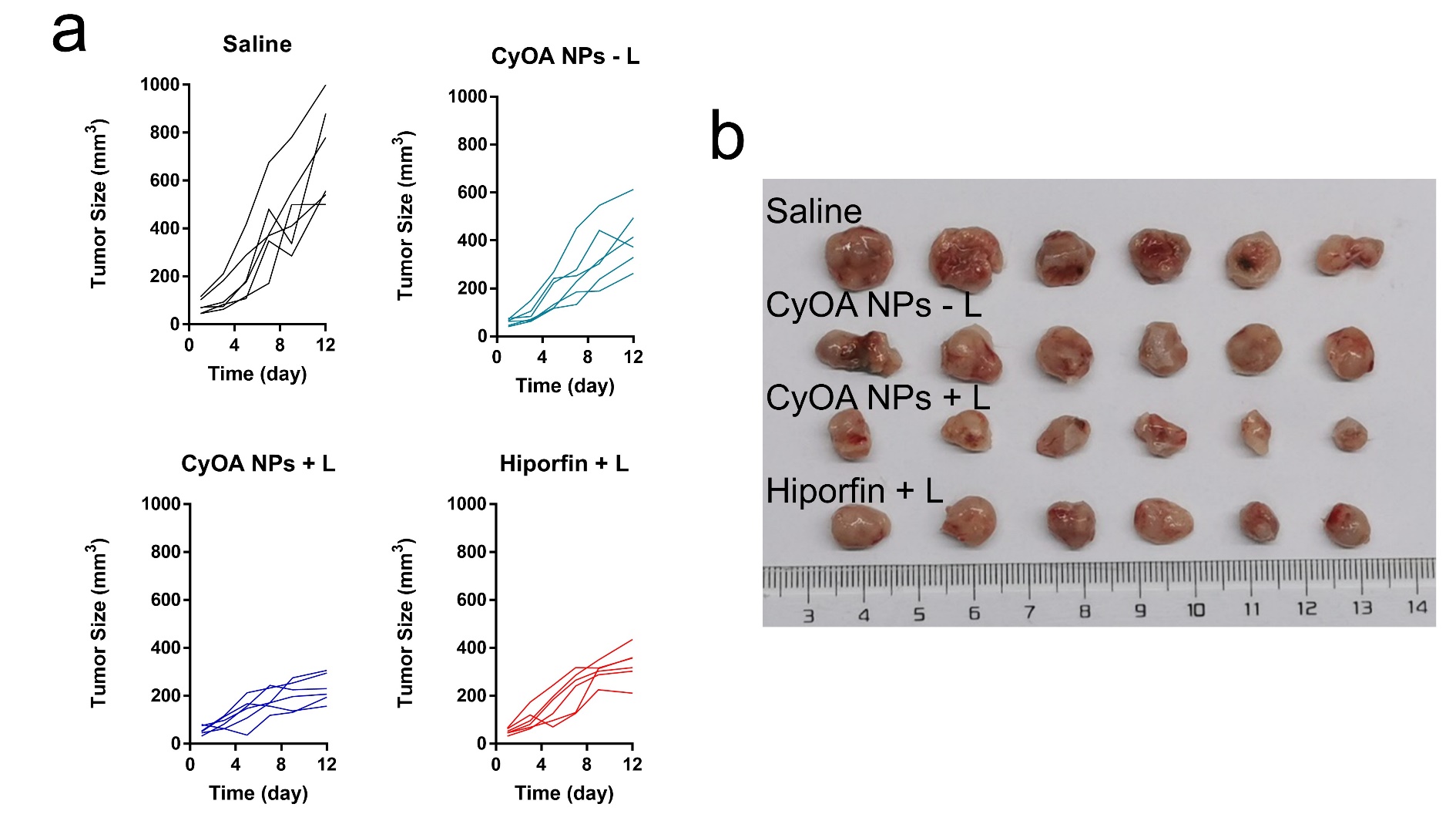
**

**Figure S14.** CyOA NPs suppress tumor growth in subcutaneous 4T1 tumor model. a. Measurement of tumor volume for each mouse. b. Photographs of excised tumors.

**
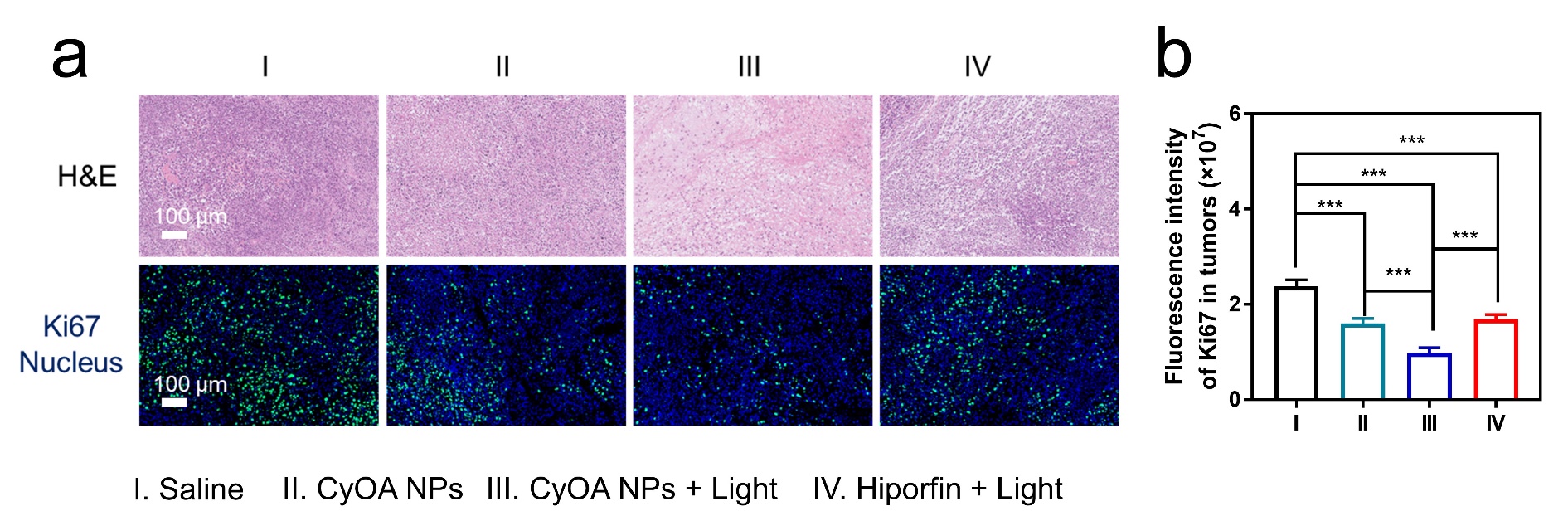
**

**Figure S15.** a. H&E staining and Ki67 fluorescence staining of tumor sections at the end of *in vivo* antitumor experiment growth in subcutaneous 4T1 tumor model. Scale bars: 100 μm. b. Quantification of proliferation indexes based on fluorescence intensity in Ki67 stained tumor sections. Data represent mean ± SEM, n = 15; statistical significance was calculated by t-test; ***, *p<0.001*.

**
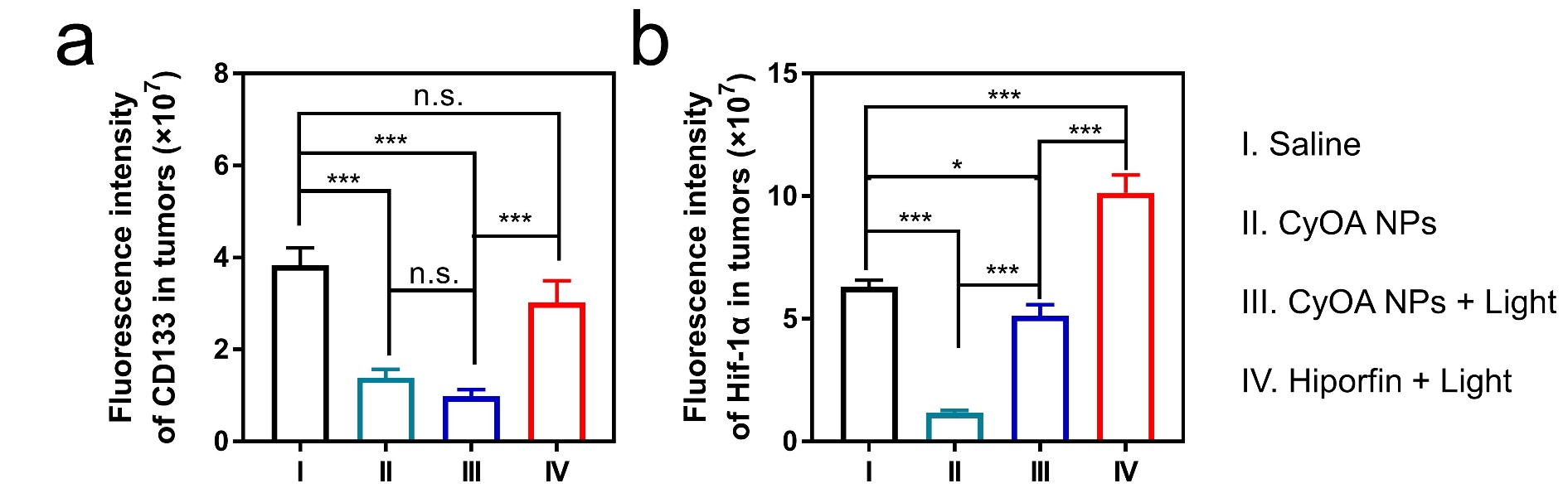
**

**Figure S16.** Quantification of CD133 (a) and Hif-1α (b) levels based on fluorescence intensity in CD133 and Hif-1α stained tumor sections, respectively. Data represent the mean ± SEM, n = 15; statistical significance was calculated by t-test; n.s., not significance, *, *p<0.05*, ***, *p<0.001*.

**
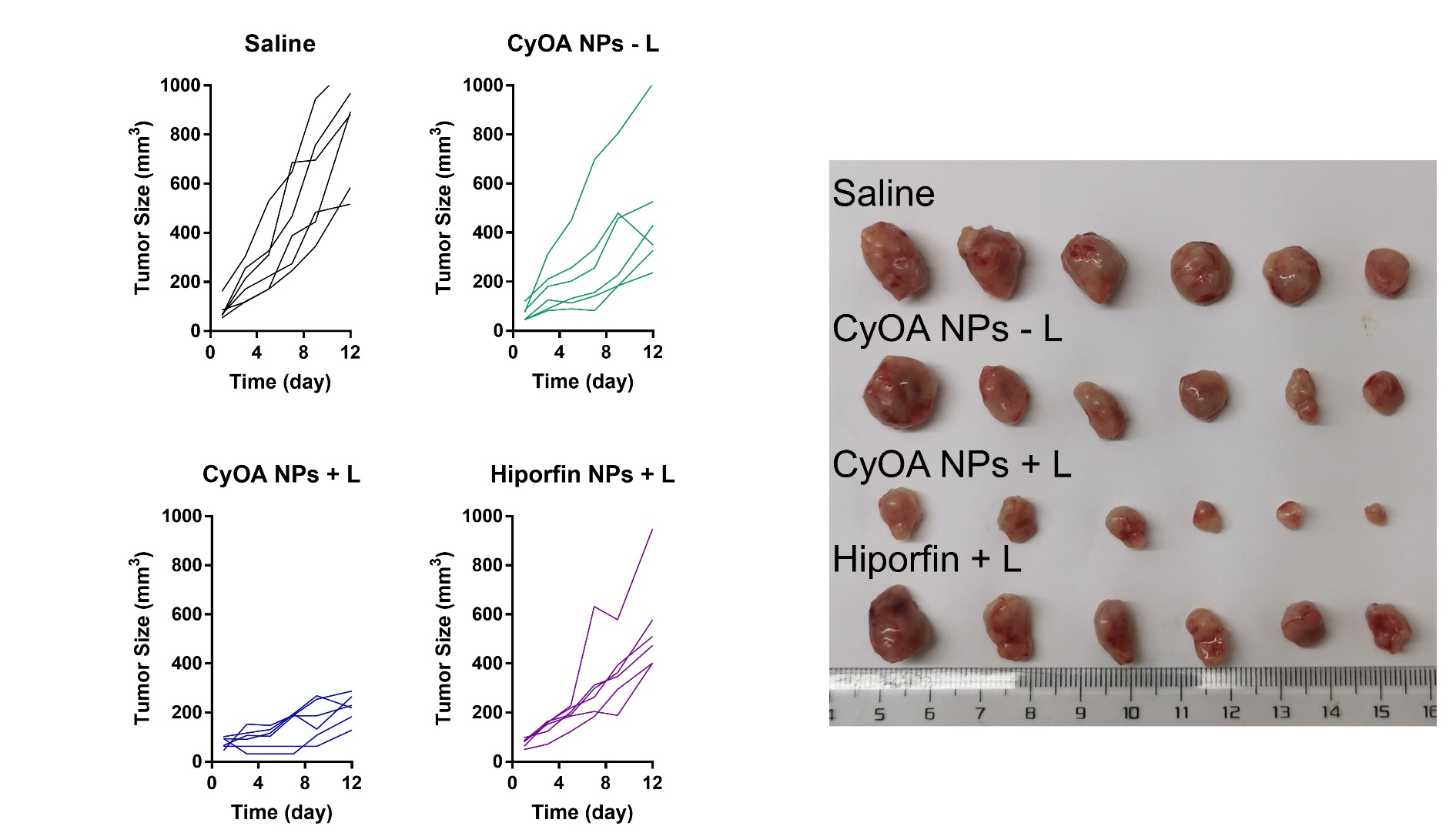
**

**Figure S17.** CyOA NPs suppress tumor growth in subcutaneous BCSCs tumor model. a. Measurement of tumor volume for each mouse. b. Photographs of excised tumors.

**
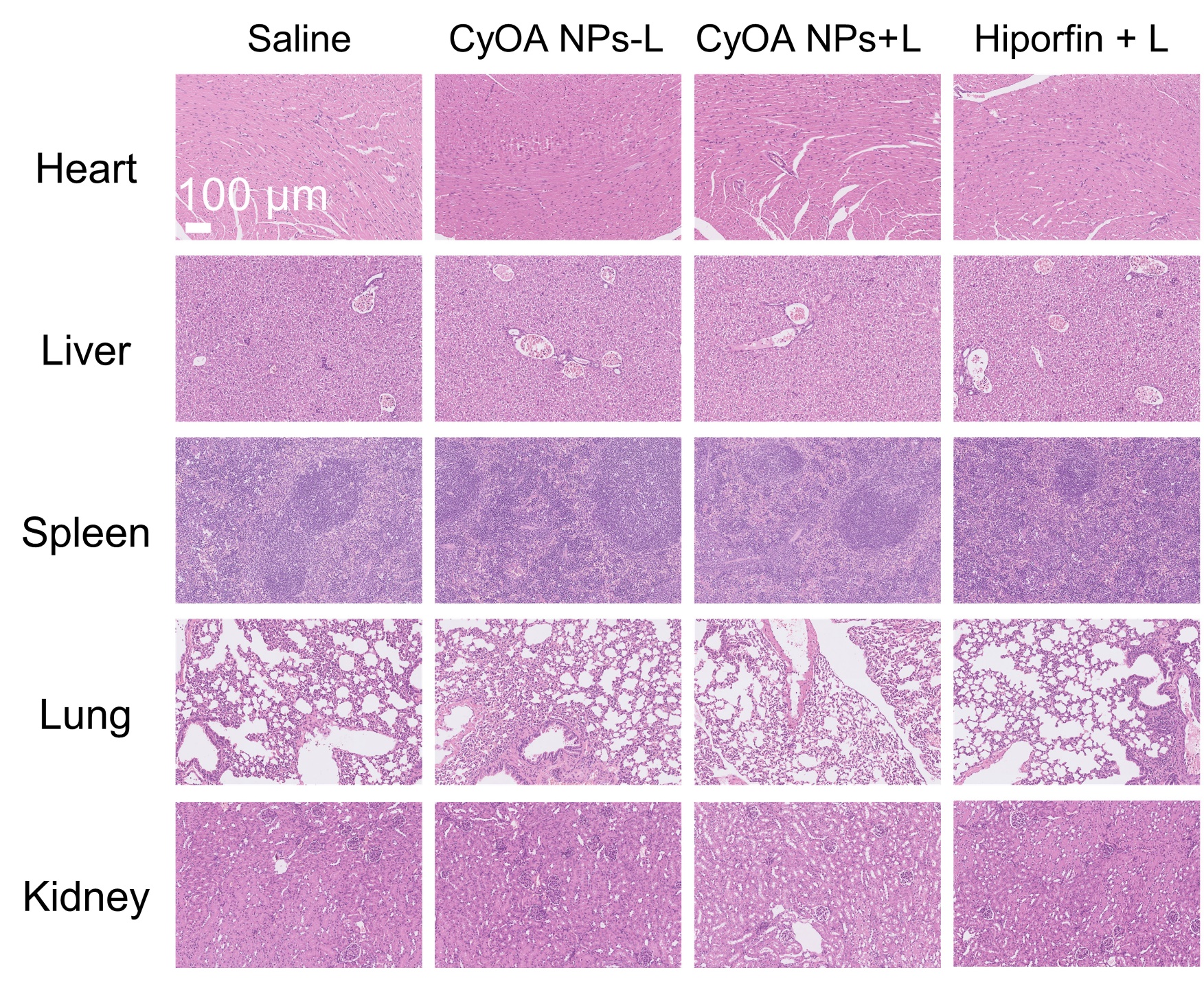
**

**Figure S18.** H&E staining of main organs after different treatments. The scale bar is 100 μm.

**
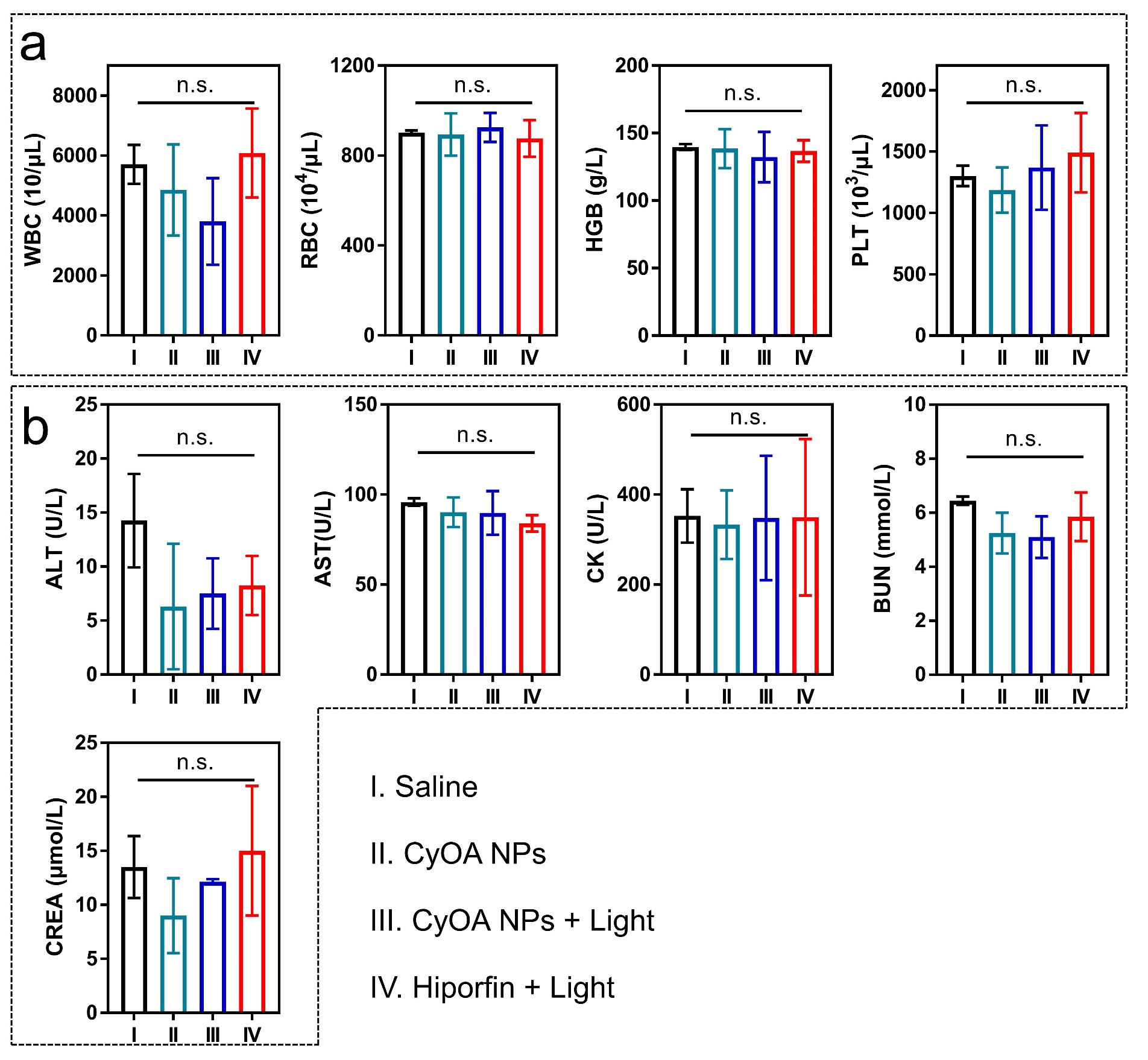
**

**Figure 19.** Routine blood (a) and blood biochemistry (b) analyses of mice in different groups. Data represent mean ± SEM, n = 5; statistical significance was calculated by t-test; n.s., not significance.

**Table S1.** Phototoxicity of SO_3_-CyOA NPs and CyOA NPs on 4T1 cells (normoxic and hypoxic) and BCSCs. Data represent the mean ± SEM (n = 3).

| Cells | IC_50_ (μM) | |
| --- | --- | --- |
|  | SO_3_-CyOA NPs + L | CyOA NPs + L |
| 4T1 (21% O_2_) | 45.7 ± 2.3 | 1.9 ± 0.1 |
| 4T1 (1% O_2_) | 79.3 ± 2.8 | 2.1 ± 0.2 |
| BCSCs | 70.6 ± 3.1 | 1.4 ± 0.2 |

**Table S2.** Pharmacokinetic parameters of CyOH and CyOA NPs after intravenous administration. Data represent the mean ± SEM (n = 3).

| Parameters | CyOH | CyOA NPs |
| --- | --- | --- |
| Dosage (μmol/kg) | 5.0 | 5.0 |
| C_0_ (μM) | 1.15 ± 0.1 | 11.6 ± 0.3^[a]^ |
| V (mL/kg) | 4326.3 ± 203.4 | 432.4 ± 12.3^[a]^ |
| CL (mL/h) | 2421.8 ± 171.3 | 145.2 ± 19.6^[a]^ |
| t_1/2_ (h) | 1.25 ± 0.1 | 2.1 ± 0.2 |
| AUC_(0-8 h)_(h·μM) | 2.04 ± 0.1 | 32.4 ± 3.5^[a]^ |

[a], *p* < 0.001 compared with CyOH. C_0_, maximum plasma concentration. t_1/2_, half life time. CL, clearance rate. AUC, area under curve.
